# DNA methylation-driven genes for constructing diagnostic, prognostic, and recurrence models for hepatocellular carcinoma

**DOI:** 10.7150/thno.31155

**Published:** 2019-09-25

**Authors:** Junyu Long, Peipei Chen, Jianzhen Lin, Yi Bai, Xu Yang, Jin Bian, Yu Lin, Dongxu Wang, Xiaobo Yang, Yongchang Zheng, Xinting Sang, Haitao Zhao

**Affiliations:** 1 Department of Liver Surgery, Peking Union Medical College Hospital, Chinese Academy of Medical Sciences & Peking Union Medical College, Beijing, China; 2 Department of Cardiology, Peking Union Medical College Hospital, Chinese Academy of Medical Sciences and Peking Union Medical College, Beijing, China.; 3 Shenzhen Withsum Technology Limited, Shenzhen, China

**Keywords:** DNA methylation-driven genes, hepatocellular carcinoma, diagnosis, prognosis, recurrence

## Abstract

In this study, we performed a comprehensively analysis of gene expression and DNA methylation data to establish diagnostic, prognostic, and recurrence models for hepatocellular carcinoma (HCC).

**Methods**: We collected gene expression and DNA methylation datasets for over 1,200 clinical samples. Integrated analyses of RNA-sequencing and DNA methylation data were performed to identify DNA methylation-driven genes. These genes were utilized in univariate, least absolute shrinkage and selection operator (LASSO), and multivariate Cox regression analyses to build a prognostic model. Recurrence and diagnostic models for HCC were also constructed using the same genes.

**Results**: A total of 123 DNA methylation-driven genes were identified. Two of these genes (SPP1 and LCAT) were chosen to construct the prognostic model. The high-risk group showed a markedly unfavorable prognosis compared to the low-risk group in both training (HR = 2.81; P < 0.001) and validation (HR = 3.06; P < 0.001) datasets. Multivariate Cox regression analysis indicated the prognostic model to be an independent predictor of prognosis (P < 0.05). Also, the recurrence model successfully distinguished the HCC recurrence rate between the high-risk and low-risk groups in both training (HR = 2.22; P < 0.001) and validation (HR = 2; P < 0.01) datasets. The two diagnostic models provided high accuracy for distinguishing HCC from normal samples and dysplastic nodules in the training and validation datasets, respectively.

**Conclusions**: We identified and validated prognostic, recurrence, and diagnostic models that were constructed using two DNA methylation-driven genes in HCC. The results obtained by integrating multidimensional genomic data offer novel research directions for HCC biomarkers and new possibilities for individualized treatment of patients with HCC.

## Introduction

Hepatocellular carcinoma (HCC) is the most common type of liver cancer, the third leading cause of cancer-related deaths, and a major aggressive malignancy worldwide 1, 2. HCC is a multistep and complex illness involving a series of genetic and epigenetic alterations that include genomic deletion, amplification, mutation, and/or insertion 3. Early diagnosis and interventional therapy, together with the development of treatment and surgical methods, have led to significant progress for treating this cancer. However, the vast majority of patients with HCC are diagnosed at an advanced stage with unfavorable overall survival 4, 5. Hence, a better understanding of HCC functional pathways and molecular mechanisms, as well as the development of crucial novel biomarkers for early diagnosis and prediction of prognosis and recurrence, is urgently needed 6.

Epigenetic alterations are universally recognized as inherited modifications that affect gene expression, DNA methylation, noncoding DNA, and histone acetylation 7. DNA methylation serves as a major epigenetic modification that is involved in the transcriptional regulation of genes and maintains the stability of the genome. Various cancers have a special deregulation signature characterized by aberrant DNA methylation 8, which regulates expression of many tumor-associated genes and is critical for tumor development. Methylation changes, which include oncogene hypomethylation and tumor suppressor gene hypermethylation, are considered crucial events in carcinogenesis, including HCC 9-11. Therefore, detecting DNA methylation-driven genes and understanding the molecular details associated with these genes might help elucidate the pathogenesis and molecular mechanisms of HCC. In recent years, several efforts to detect cancer methylation using genome-wide techniques have shown that a large number of genes exhibit abnormal DNA methylation profiles in cancer 12, 13. Furthermore, these changes can be utilized to classify cancer subtypes and predict cancer outcomes 13, 14. Overall, identification of genes that act as a “driver” through gene silencing mediated by DNA methylation in the initiation, maintenance, and development of cancers as well as those that act as a “passenger” only during tumorigenic processes may be highly beneficial for developing optimal targeted epigenetic therapies 15. Nevertheless, distinguishing driver and passenger genes has proven to be quite difficult because of the numerous genes differentially methylated in human cancers 16.

Previously, analysis of profiling arrays has demonstrated that HCC pathogenesis is a complex biological process that involves genetic and epigenetic changes 17, and DNA hypermethylation is an early event in the development of HCC 18. One meta-analysis offered empirical evidence that abnormal promoter methylation of suppressor of cytokine signaling 1 (SOCS1) might lead to HCC pathogenesis 19. It has also been reported that retinol metabolism genes and serine hydroxymethyltransferase 1 are epigenetically regulated via promoter DNA methylation in alcohol-related HCC 20. However, most studies have focused mainly on either methylation or gene expression data and have not conducted combined analyses. In general, the lack of a comprehensive understanding of the cellular and molecular mechanisms driving HCC restricts treatment strategies. However, combining methylation microarray and gene expression data will allow for methylation and expression to be detected simultaneously, enabling more accurate identification of the biological characteristics of cancers 21. In the present study, transcriptomic and DNA methylation profiles were utilized to identify DNA methylation-driven genes and to generate three prediction models for HCC. Our findings will help further improve molecular diagnoses and individualized therapies for HCC.

## Methods

### Patients and samples

A total of 421 RNA-sequencing profiles (371 HCC samples and 50 nontumor samples), 430 DNA methylation profiles (380 HCC samples and 50 nontumor samples) and the corresponding clinical information of HCC patients were acquired from The Cancer Genome Atlas (TCGA) (up to September 1, 2018) (Table S1). Among 380 HCC samples for which DNA methylation data were available, 371 HCC samples included both RNA-sequencing data and paired DNA methylation data (Table S1). Among 371 TCGA HCC samples, 365 included overall survival time and survival statuses, and 317 included disease-free survival time and recurrence statuses. HCC gene expression data from TCGA were acquired with the Illumina HiSeq 2000 RNA Sequencing platform, and DNA methylation data were obtained with the Illumina Infinium Human Methylation 450 platform. The average DNA methylation value for all CpG sites in the promoters (transcription start sites (TSS) 1500 and TSS200) of a gene was calculated as the DNA methylation value for that gene. The GSE14520 microarray dataset including gene expression profiles (225 HCC samples and 220 nontumor samples) and the associated clinical characteristics, the GSE6764 microarray dataset including gene expression profiles (35 HCC samples and 17 dysplastic nodules samples), the GSE89377 microarray dataset including gene expression profiles (40 HCC samples and 22 dysplastic nodules samples), GSE56588 microarray dataset including DNA methylation profiles (214 HCC samples), and GSE63898 microarray dataset including gene expression profiles (214 HCC samples) analyzed in current research were acquired from the Gene Expression Omnibus (GEO) database 22. Among 225 HCC samples in the GSE14520 dataset, 221 included overall survival (OS) time and survival statuses, and 221 included disease-free survival time and recurrence statuses. Data were utilized according to the data access policy of GEO and TCGA. All analyses were conducted in accordance with relevant regulations and guidelines.

### Screening for differentially expressed genes (DEGs) in HCC

To search for genes for critical HCC development, we identified DEGs between 371 HCC samples and 50 nontumor samples from TCGA utilizing the “edgeR” R package 23. To select genes for further analysis, the false discovery rate (FDR) < 0.05 and |log_2_ fold change (FC)| > 1 were utilized as cutoff criteria.

### Comprehensive analysis of gene expression and DNA methylation

In the current research, the MethylMix package in R was utilized for analysis that integrated DNA methylation data for 371 HCC samples and 50 nontumor samples and paired gene expression data for 371 HCC samples to identify DNA methylation events that significantly affect the expression of the corresponding gene, indicating that the gene is a DNA methylation-driven gene 24. The MethylMix analysis included three parts. First, the correlation between the methylation data and paired gene expression data of DEGs for 371 HCC samples was determined to identify DNA methylation events that lead to changes in gene expression, and only genes that passed the correlation filter were chosen for further analysis. Second, a beta mixture model was utilized to define a methylation state across a large number of patients, precluding the need for an arbitrary threshold. Third, the Wilcoxon rank sum test was utilized to compare DNA methylation states between the 371 HCC samples and 50 corresponding nontumor samples 24. Multiple testing was performed with a q value of 0.05 as the cutoff.

### Generation and validation of the predictive model

Univariate, least absolute shrinkage and selection operator (LASSO) and multivariate Cox regression analyses were utilized to evaluate relationships between the expression of the DNA methylation-driven gene and prognosis and to identify independent DNA methylation-driven genes that were significantly associated with prognosis for the dataset from TCGA. A DNA methylation-driven gene-based risk score prediction model was established through linear combination of the expression levels of independent DNA methylation-driven genes using coefficients from multivariate Cox regression as the weights. Based on the DNA methylation-driven gene-based risk score prediction model, HCC patients were stratified into low-risk and high-risk groups with the optimal risk score as the cutoff point. We used X-tile software to find the optimal cutoff value. The threshold for the risk score that was output from the prediction model, which was utilized for separating patients into high-risk and low-risk groups, was defined as the risk score that generated the largest value of χ² in the Mantel-Cox test. Survival differences between high-risk patients and low-risk patients were evaluated by Kaplan-Meier survival plots and then compared utilizing the log-rank test. Time-dependent receiver operating characteristic (ROC) curves were employed to measure predictive performance, and the GSE14520 dataset from the GEO database was used to validate the prognostic model.

### Independence of the predictive model score from clinicopathological features

Univariate and multivariate Cox regression analyses were performed to determine whether the predictive power of the predictive model may be independent of other clinical features (including alpha-fetoprotein (AFP), age, weight, sex, histologic grade, inflammation, pathologic stage, vascular tumor invasion, and family history) of HCC patients.

### Building and validating the nomogram

We included each independent predictive factor selected by the multivariate Cox regression analysis to generate a nomogram using the “rms” package. Validation steps, which included calibration and discrimination, were then carried out. A concordance index (C-index) was utilized to calculate the nomogram discrimination via a bootstrap method with 1000 resamples, and calibration curves were graphically evaluated by plotting the observed rates against the probabilities predicted by the nomogram, whereby the 45° line represented the best prediction.

### External validation of gene expression levels of DNA methylation-driven genes

We also attempted to verify the expression pattern of DNA methylation-driven genes in TCGA; hence, the expression of these genes based on the GSE14520 dataset was obtained for further analysis. Differential expression patterns of the DNA methylation-driven genes between the HCC and nontumor samples were analyzed using the Wilcoxon signed-rank test. A p-value of less than 0.05 was considered significant, and all statistical tests were two-sided.

### Cell culture

The HCC cell line HepG2 was purchased from ATCC (ATCC® HB-8065™) and maintained in Minimum Essential Medium (Gibco, Cat No. 11095-080) at 37 °C supplemented with 10% fetal calf serum (Hyclone, Cat No. SH30084.03) in a humidified atmosphere containing 5% CO_2_.

### Treatment with 5-aza-2'-deoxycytidine (DAC)

HepG2 cells in culture were treated with 5 μM/L 5-aza-2'-deoxycytidine (DAC) (Sigma-Aldrich, Cat No. A3656-5MG) for 120 h, and the medium was changed every day due to DAC instability. For experiments involving DAC treatment, dimethyl sulfoxide (DMSO) was utilized as the control treatment. The cells were harvested for extraction of genomic DNA and total RNA for analysis of DNA methylation and gene expression.

### DNA extraction and analysis of DNA methylation

Sequencing primers were designed to include fragments with CpG sites within 0.5 kb of the transcription start site (Table S2). Methylation levels equal to or lower than 15% were considered indistinguishable from background, and a methylation level of 15% or higher was considered positive for methylation. We extracted genomic DNA from cancer cells using E.Z.N.A.®Tissue DNA Kit (Omega, Cat No. D3396-01) and treated the DNA samples with sodium bisulfite using EZ DNA Methylation-Gold™ Kit (ZYMO, Cat no. D5006). Bisulfite pyrosequencing was carried out to verify bioinformatics results for tissue samples. For pyrosequencing, the treated DNA samples were amplified by PCR and fragmented. The processed samples were then precipitated, suspended, and genotyped using the Pyro Mark Q96 system (Qiagen, Hilden, Germany, Cat no. 979002).

### Validation of mRNAs using quantitative real-time polymerase chain reaction (qRT-PCR)

Table S3 shows the primers used for qRT-PCR. Total RNA was extracted from cultured cancer cells using the Trizol reagent (Thermofisher, Cat No.15596026) in accordance with the manufacturer's instructions. The cDNA reverse transcription kit (TOYOBO, Cat No. FSQ-101) was used to reverse transcribe RNA, and the SYBR Green PCR kit (Applied Biosystems, Cat. No. 4368708) was utilized to amplify the resulting cDNA. The samples were detected with QuantStudio 5 Real-Time PCR System (Applied Biosystems; Thermo Fisher Scientific). Each experiment was conducted at least three times. The 2^-ΔΔCt^ method was adopted to calculate expression of genes relative to the housekeeping gene GAPDH.

## Results

### Identification of DEGs in HCC

mRNA expression profiles (level 3 data) in HCC tissues (n=371) and nontumor tissues (n=50) were obtained from TCGA (Table S1). Using the threshold of FDR < 0.05 and |log_2_ FC| > 1, a total of 9,219 DEGs (7,734 upregulated and 1,485 downregulated) were selected for subsequent analysis.

### Identification of DNA methylation-driven genes in HCC

**Figure 1:**
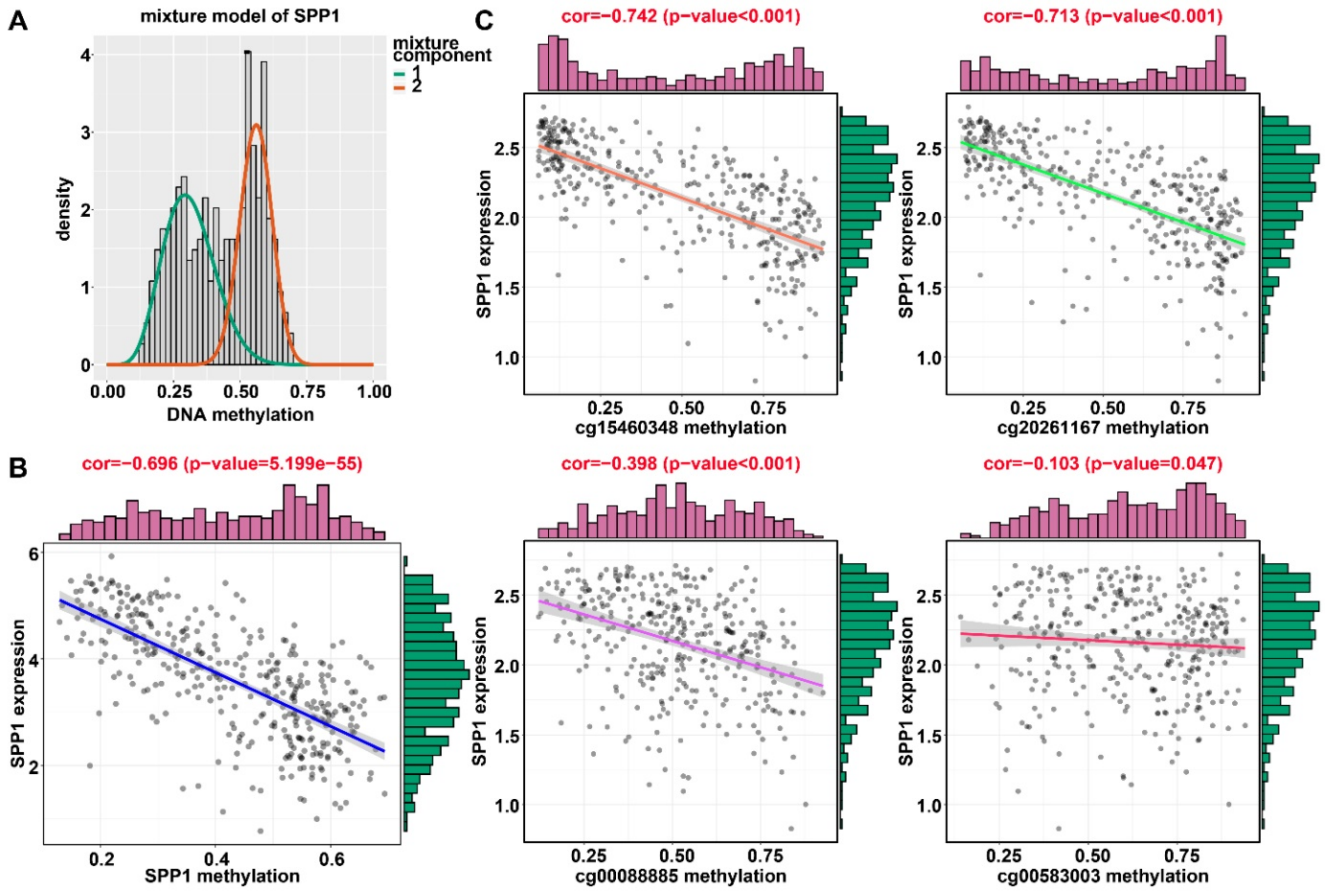
** Regression analysis between gene expression and DNA methylation of SPP1 in the training dataset from TCGA.** (A) Mixture models for SPP1. The horizontal black bar indicates the distribution of methylation values in normal samples. The histogram illustrates the distribution of methylation in tumor samples (signified as beta values, where higher beta values denote greater methylation). (B) Regression analysis between gene expression and DNA methylation of SPP1. (C) Regression analysis between gene expression and DNA methylation of CpGs in the SPP1 promoter. The vertical axis represents methylation of the DNA methylation-driven gene, and the horizontal axis denotes mRNA expression of the DNA methylation-driven gene. The right and upper edges are histograms of DNA methylation and gene expression, respectively.

**Figure 2:**
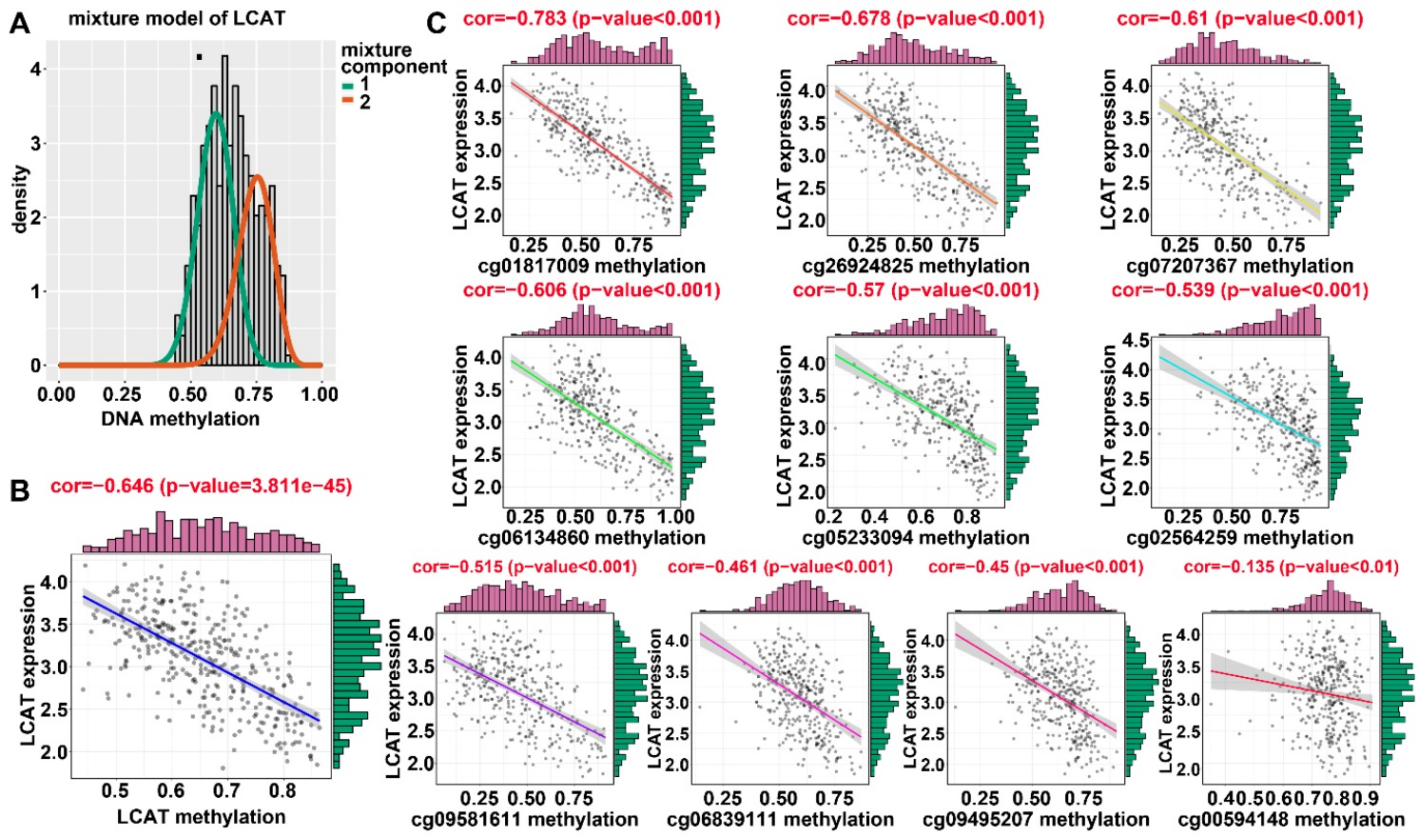
** Regression analysis between gene expression and DNA methylation of LCAT in the training dataset from TCGA.** (A) Mixture models for LCAT. The horizontal black bar indicates the distribution of methylation values in normal samples. The histogram illustrates the distribution of methylation in tumor samples (signified as beta values, where higher beta values denote greater methylation). (B) Regression analysis between gene expression and DNA methylation of LCAT. (C) Regression analysis between gene expression and DNA methylation of CpGs in the LCAT promoter. The vertical axis represents methylation of the DNA methylation-driven gene, and the horizontal axis denotes mRNA expression of the DNA methylation-driven gene. The right and upper edges are histograms of DNA methylation and gene expression, respectively.

To identify DNA methylation-driven genes in HCC, gene expression and DNA methylation data for 9,219 DEGs from 792 clinical samples (DNA methylation data of 371 HCC samples and 50 nontumor samples and the paired gene expression data for 371 HCC samples) from TCGA were included in the MethylMix analysis. A total of 123 DNA methylation-driven genes were screened. Of these genes, 77 were hypermethylated and 46 hypomethylated (Figures 1A and 2A) (Table S4). The inclusion criteria were an FDR < 0.05 between the hyper- and hypomethylation groups and the correlation between DNA methylation and gene expression of less than -0.3. We then investigated the relationship between expression of 123 DNA methylation-driven genes and prognosis employing univariate Cox proportional hazard regression analysis using 365 HCC samples with OS time and survival status. Among the 123 DNA methylation-driven genes included in the analysis, 51 were statistically significant (P < 0.05). LASSO is a penalized regression method that uses an L1 penalty to shrink regression coefficients toward zero, thereby eliminating a number of variables based on the principle that fewer predictors are selected when the penalty is larger 25. Thus, seed genes with nonzero coefficients were regarded as potential prognostic predictors. Based on 1000 iterations of Cox LASSO regression with 10-fold cross-validation using the R package glmnet, the seed genes were shrunk into multiple-gene sets. Genes with nonzero coefficients were considered potential prognostic genes. The higher the nonzero coefficients that occurred in 1000 iterations of Cox LASSO regression, the stronger was the ability of this gene to predict prognosis 26. The 51 selected DNA methylation-driven genes were analyzed by 1000 iterations of Cox LASSO regression to reduce the number further.

**Figure 3:**
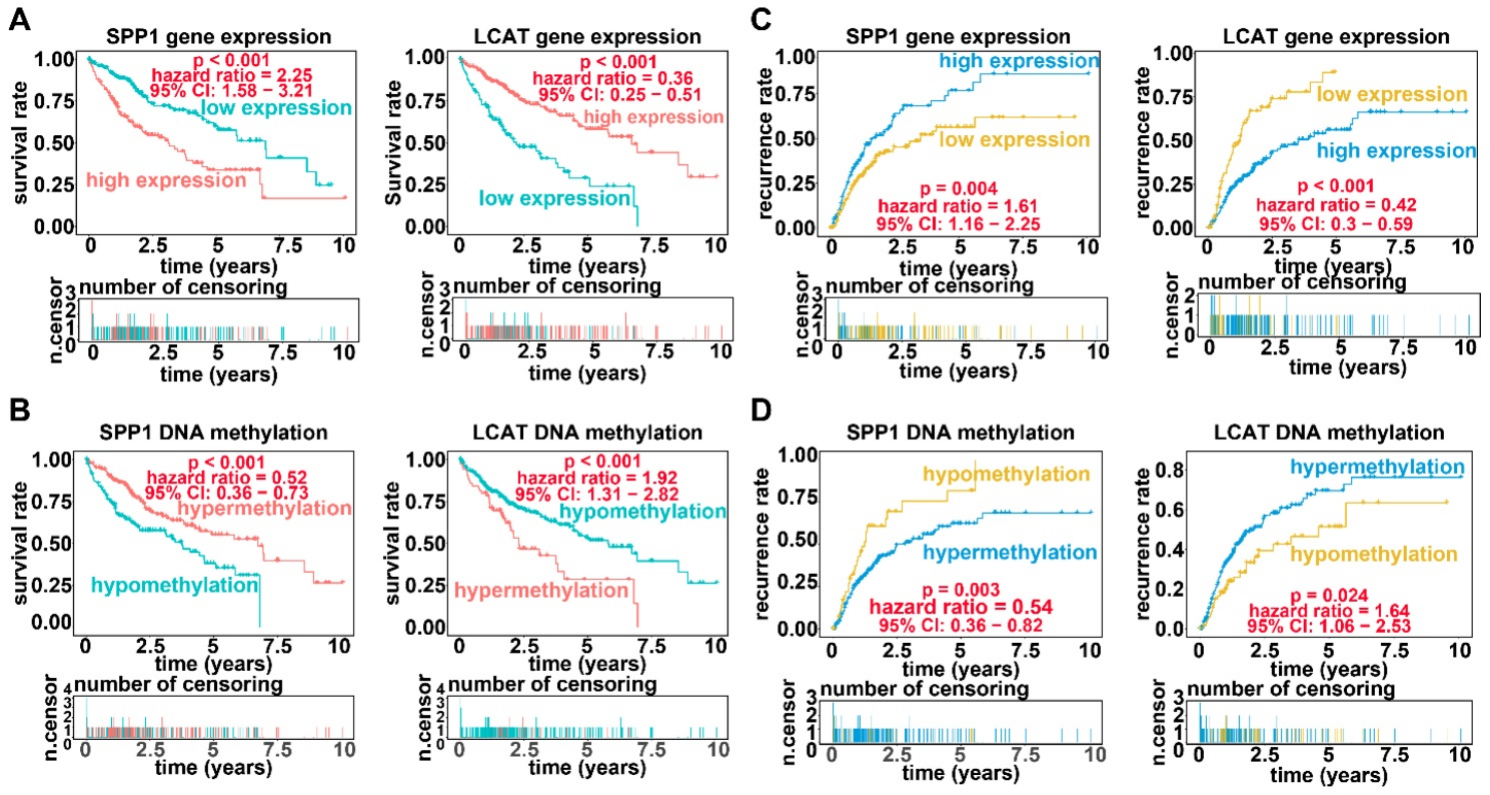
** Survival analysis and recurrence analysis for SPP1 and LCAT.** (A) Survival analysis based on gene expression data for SPP1 and LCAT. The horizontal axis indicates the survival time, and the vertical axis indicates the survival rate. (B) Recurrence analysis based on expression data for SPP1 and LCAT. The horizontal axis indicates the disease-free survival time, and the vertical axis indicates the recurrence rate. (C) Survival analysis based on DNA methylation data for SPP1 and LCAT. (D) Recurrence analysis based on DNA methylation data for SPP1 and LCAT.

Applying LASSO analysis, in which the selected genes were required to appear 1000 times out of 1000 repetitions, two DNA methylation-driven genes, secreted phosphoprotein 1 (SPP1) and lecithin-cholesterol acyltransferase (LCAT), were selected as prognostic genes (Table S5). Correlation analyses showed that gene expression had a significantly negative correlation with DNA methylation of SPP1 and LCAT (Figures 1B and 2B). We also explored which specific CpGs in the promoters may drive expression of both genes. Our results showed a significantly negative correlation between all CpGs in the SPP1 promoter and SPP1 mRNA expression, whereas 9 of 13 CpGs in the LCAT promoter were significantly negatively corelated with LCAT mRNA expression (Figures 1C and 2C) (Table S6). We then performed survival and recurrence analyses using the DNA methylation and gene expression data for SPP1 and LCAT in the training dataset from TCGA. High gene expression and DNA hypomethylation of SPP1 were significantly associated with a poor prognosis and high recurrence rate, and low gene expression and DNA hypermethylation of LCAT had a significant association with a poor prognosis and low recurrence rate, further demonstrating a negative regulatory relationship between DNA methylation and gene expression (Figure 3).

### Generation and validation of the prognostic model based on DNA methylation-driven genes

**Figure 4:**
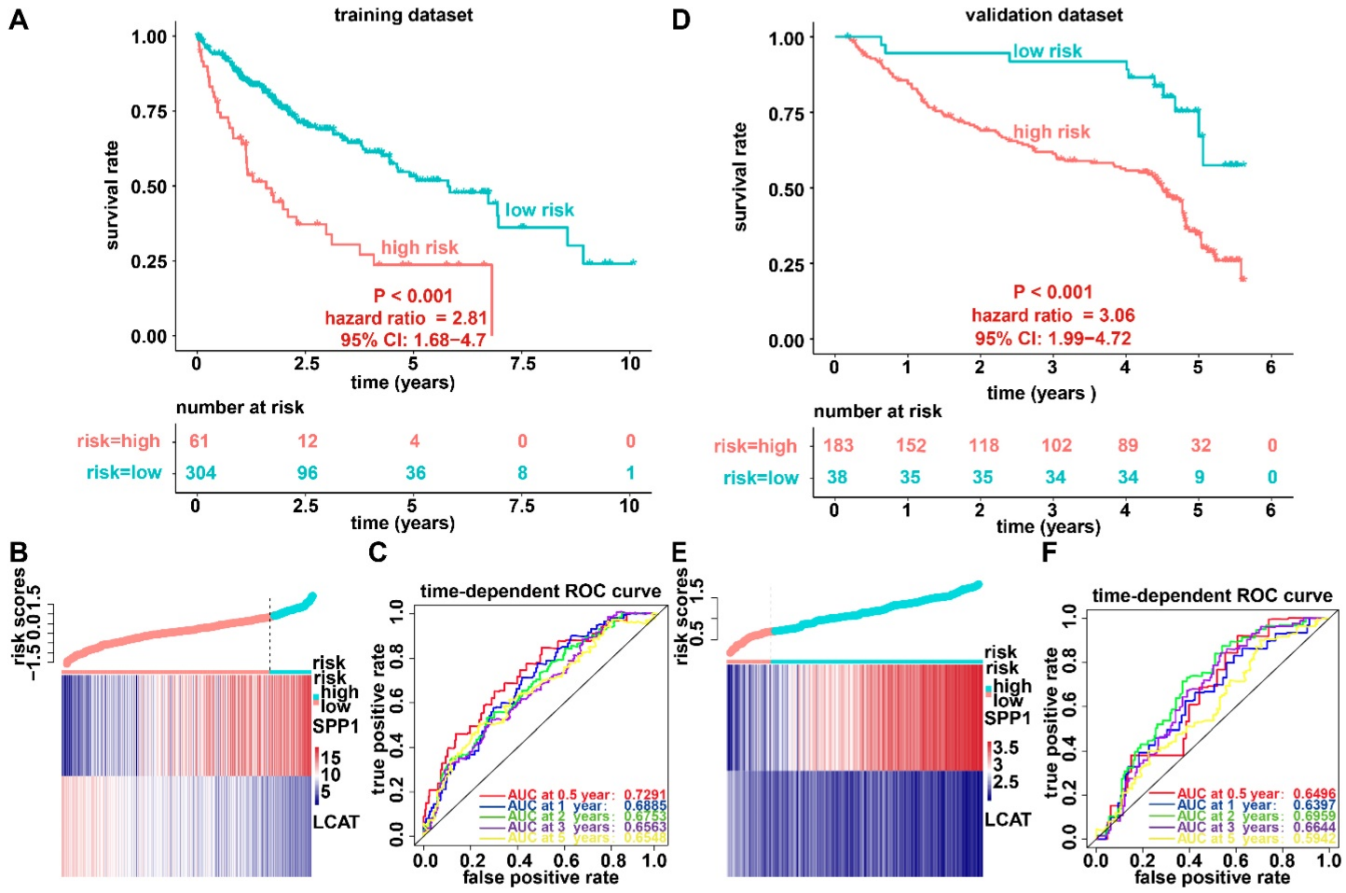
** Survival analysis, risk score distribution, and evaluation of the prognostic model for the training (A-C) and validation (D-F) datasets.** (A and D) Kaplan-Meier curve of the prognostic model. (B and E) Distribution of the expression of DNA methylation-driven genes (bottom) and risk score (upper). (C and F) Accuracy of the prognostic model in predicting survival time.

We established a prognostic model utilizing the regression coefficient from a multivariate Cox proportional hazard model. The risk score was calculated according to the formula, 0.0862 × SPP1 expression level - 0.1719 × LCAT expression level. The patients were stratified into high-risk and low-risk groups relative to the optimum cutoff point. High-risk patients showed markedly worse OS (hazard ratio, HR = 2.81, 95% confidence interval, 95% CI = 1.68-4.7, P < 0.001) than did low-risk patients (Figure 4A). Figure 4B displays gene expression and risk score distribution. We also conducted a ROC analysis to determine the specificity and sensitivity of the prognostic model. The time-dependent area under the curves (AUCs) for 0.5-, 1-, 2-, 3-, and 5-year OS rates for HCC cases with the prognostic model were 0.7291, 0.6885, 0.6753, 0.6562, and 0.6548, respectively (Figure 4C). The predictive ability of the prognostic model was further tested using 221 HCC samples with OS time and survival status in the validation dataset (GSE14520). The patients were classified into low-risk and high-risk groups utilizing the formula mentioned earlier based on the optimal cutoff value in the validation dataset. Consistent with the above findings, patients in the high-risk group in the validation set had a markedly shorter median OS than those in the high-risk group (HR = 3.06, 95% CI = 1.99-4.72, P < 0.001) (Figure 4D). Figure 4E shows the distribution of risk scores and gene expression. The time-dependent AUCs of 0.5-, 1-, 2-, 3-, and 5-year OS rates with the prognostic model for HCC cases were 0.6496, 0.6397, 0.6959, 0.6643 and 0.5942, respectively (Figure 4F).

### Establishment of a prognostic nomogram for OS prediction in HCC

**Figure 5:**
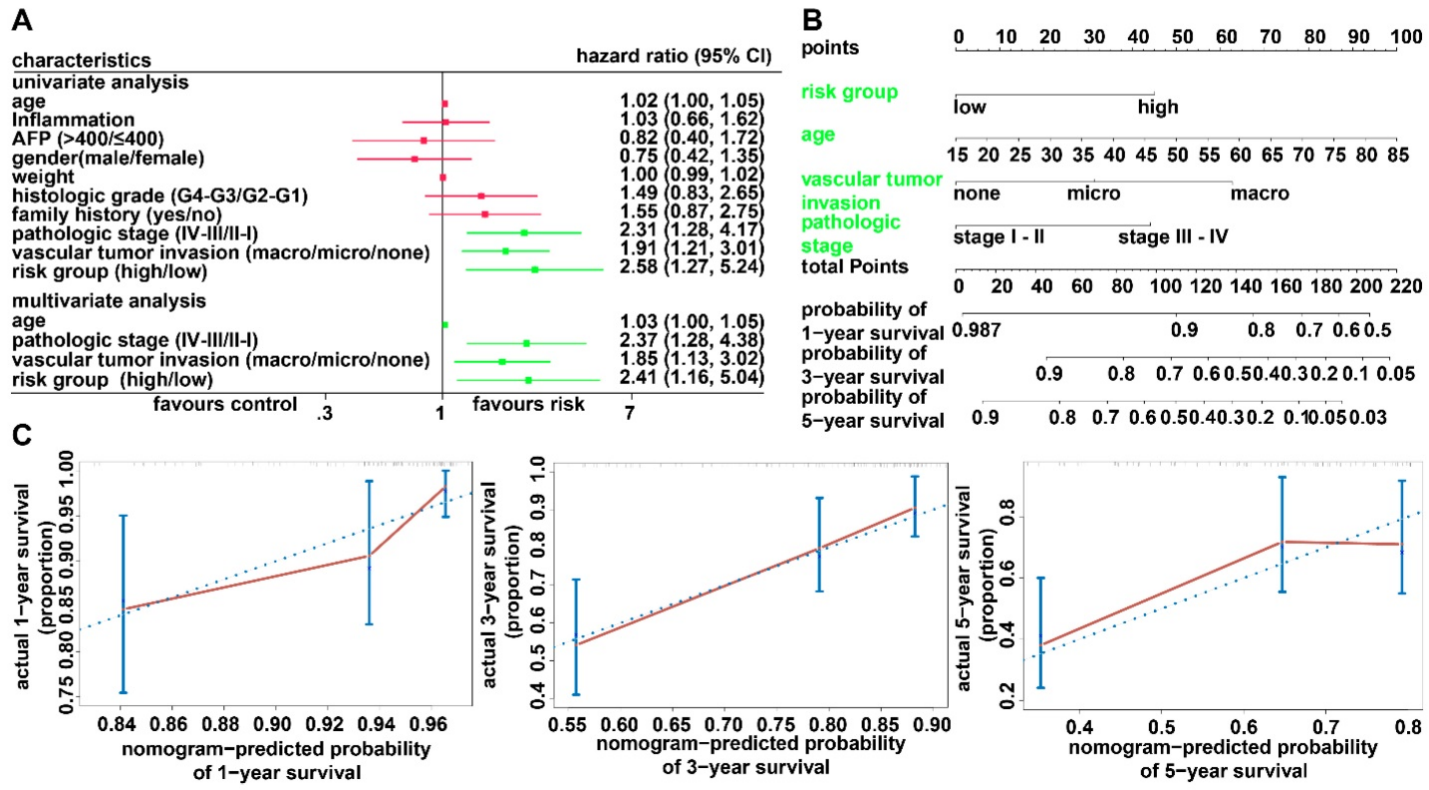
** Relationship between the prognostic model and clinicopathological characteristics.** (A) Univariate and multivariate regression analyses for the prognostic model and clinical characteristics. Green represents statistical significance; red represents no statistical significance. (B) Nomogram for predicting the probability of 1-, 3-, and 5-year survival times for patients with HCC. (C) Calibration plot of the nomogram for predicting the probability of survival at 1, 3, and 5 years.

To investigate whether the prognostic model is independent of the clinicopathological features, univariate and multivariate Cox regression analyses were conducted utilizing risk group, tumor stage, sex, and age as covariates. The analysis indicated that the prognostic model was a significant independent factor for OS (HR = 2.41, P = 0.019) (Figure 5A). To provide clinicians with a quantitative approach for predicting the individual probability of 1-, 3- and 5-year survival times, we established a prognostic nomogram integrating clinicopathological-independent risk factors and the prognostic model (Figure 5B). The C-index for the nomogram was 0.6963 (95% CI: 0.6193- 0.7733). Moreover, the calibration curves of the nomogram showed good agreement between the predicted 1-, 3-, and 5-year OS rates and actual observations (Figure 5C).

### Generation and validation of the recurrence model based on DNA methylation-driven genes

**Figure 6:**
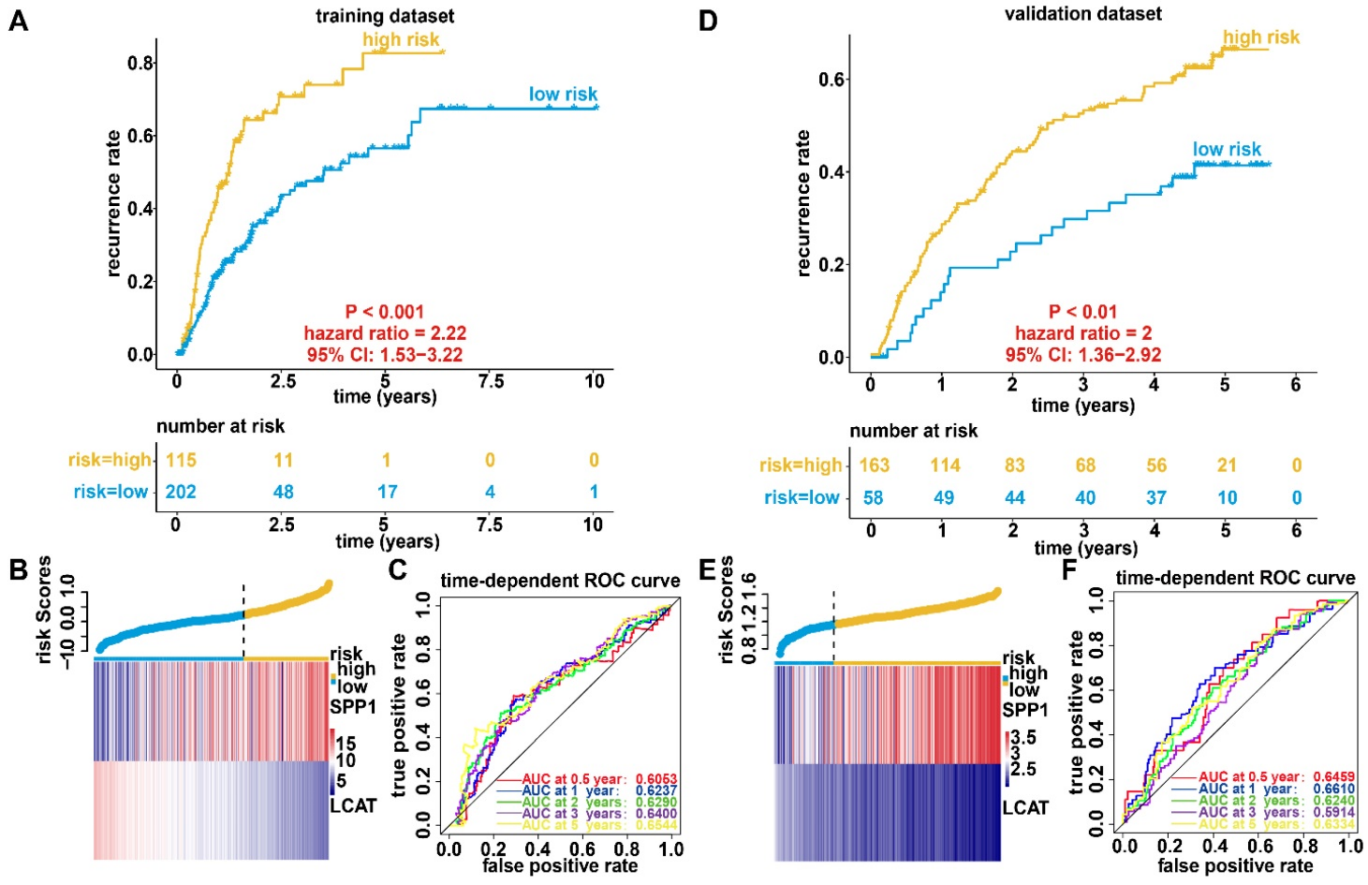
** Recurrence analysis, risk score distribution, and evaluation of the recurrence model for the training (A-C) and validation (D-F) datasets.** (A and D) Kaplan-Meier curve of the recurrence model. (B and E) Distribution of the expression of DNA methylation-driven genes (bottom) and risk score (upper). (C and F) Accuracy of the prognostic model in predicting recurrence rate.

We further constructed a recurrence model based on the two DNA methylation-driven genes utilizing regression coefficients from a multivariate Cox proportional hazards model of 317 HCC samples with disease-free survival time and recurrence status in the dataset from TCGA. The risk score was calculated as 0.0242 × SPP1 expression level - 0.1597 × LCAT expression level. High-risk patients showed a markedly higher recurrence rate (HR = 2.22, 95% CI = 1.53-3.22, P < 0.001) than did low-risk patients (Figure 6A). the gene expression and risk score distribution are presented in Figure 6B. The recurrence model achieved an AUC of 0.6053, 0.6237, 0.6290, 0.6400, and 0.6544 for 0.5-, 1-, 2-, 3-, and 5-year OS, respectively (Figure 6C). To determine the robustness of the recurrence model derived from the dataset from TCGA, we also assessed the performance of the recurrence model with 221 HCC samples with disease-free survival time and recurrence status in the validation dataset. Based on the Cox model-derived risk score, patients in the validation dataset were divided into high-risk and low-risk groups according to the optimal cutoff value (Figure 6D). Consistent with the results of the dataset from TCGA, high-risk patients exhibited a markedly higher recurrence rate than did low-risk patients (Figure 6E). Furthermore, the AUC at diverse cutoff times indicated that the predictive accuracy of the recurrence model was acceptable (Figure 6F).

### Construction of a recurrence nomogram based on the recurrence model

**Figure 7:**
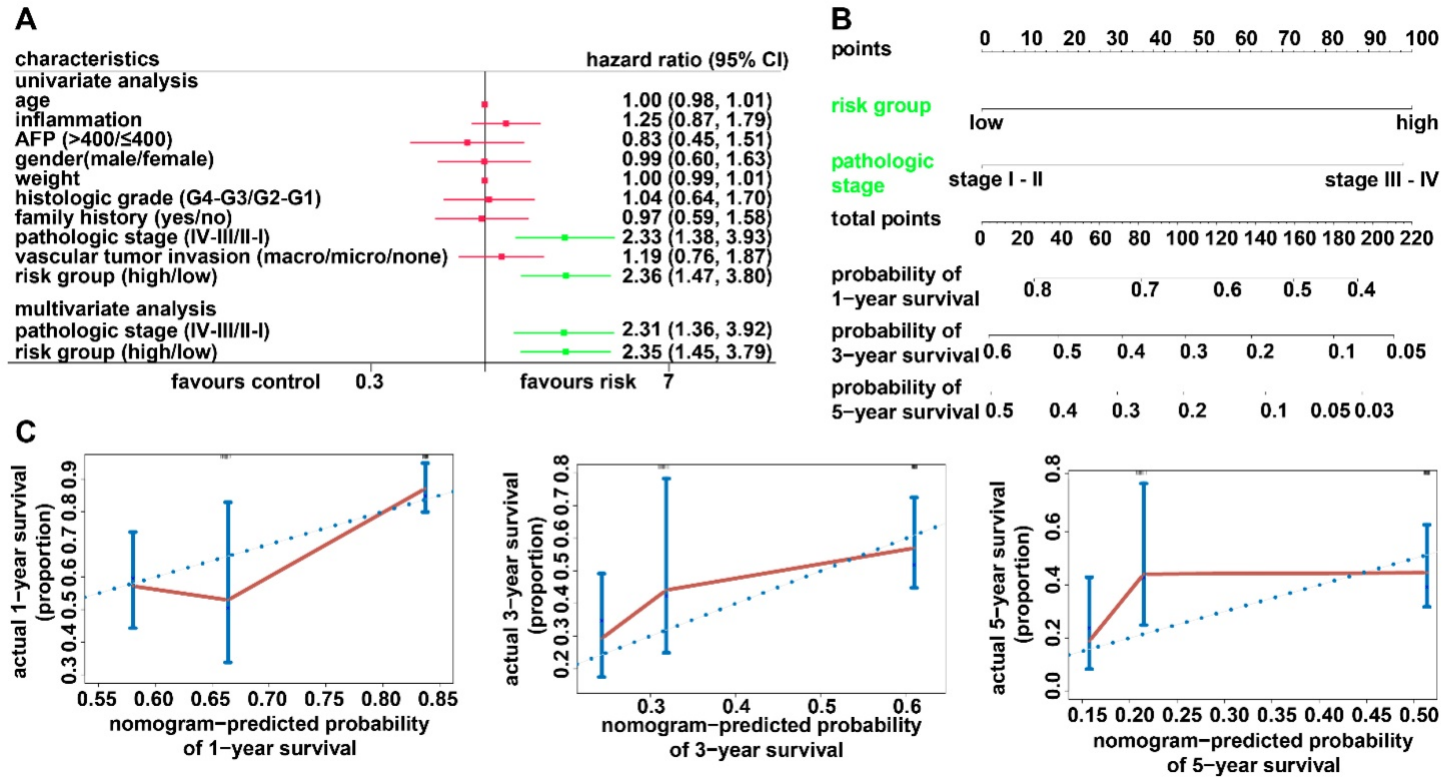
** Relationship between the recurrence model and clinicopathological characteristics.** (A) Univariate and multivariate regression analyses for the recurrence model and clinical characteristics. Green represents statistical significance, and red represents no statistical significance. (B) Nomogram for predicting the probability of 1-, 3-, and 5-year recurrence rates for patients with HCC. (C) Calibration plot of the nomogram for predicting the probability of recurrence at 1, 3, and 5 years.

Univariate and multivariate Cox regression analyses were performed to investigate whether the predictive ability of the recurrence model is independent of any other clinical factors. We observed that the recurrence model and pathology stage were significant in Cox regression analyses (Figure 7A). A recurrence nomogram was then formulated based on the two significantly independent factors (Figure 7B). The C-index was 0.6518 (95% CI, 0.5891-0.7145), indicating a favorable discrimination performance. The bias-corrected line of the calibration plot was close to the ideal curve (45° line), showing good agreement between the observation and the prediction (Figure 7C).

### Generation and validation of the diagnostic model based on two DNA methylation-driven genes

**Figure 8:**
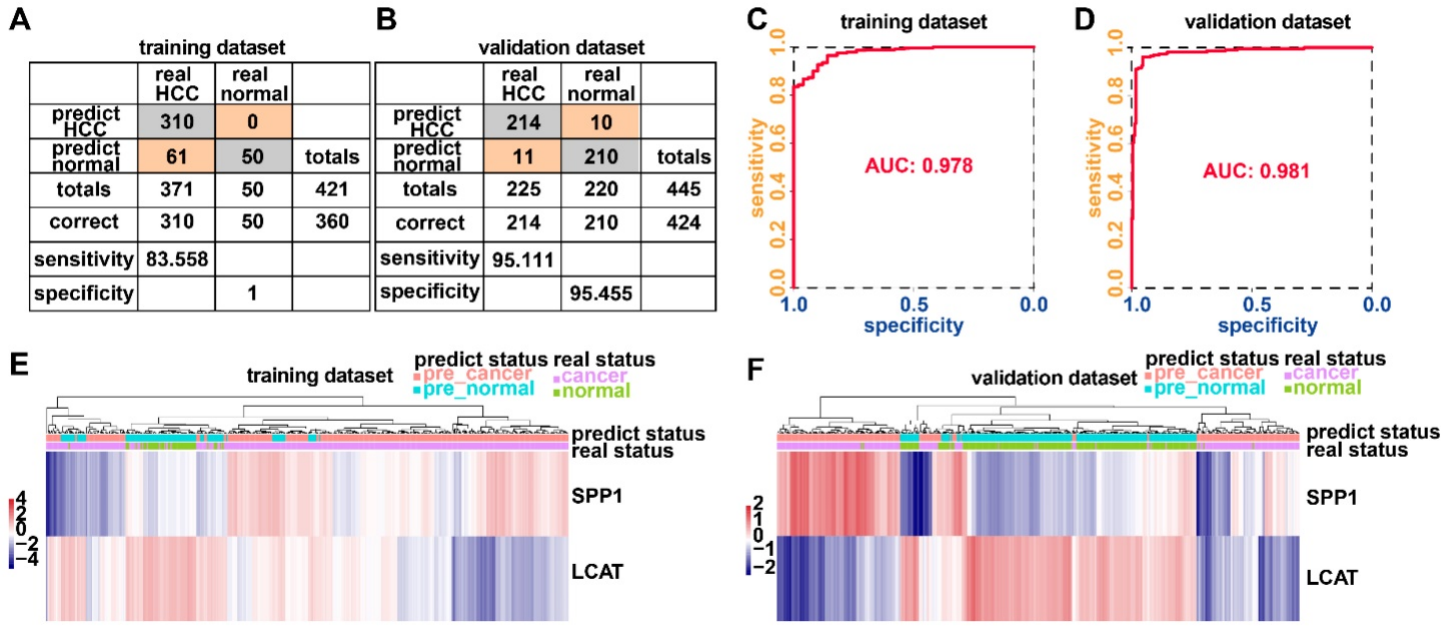
** Two DNA methylation-driven genes for distinguishing HCC from normal samples.** (A and B) Confusion matrices of binary results of the diagnostic prediction model for training (A) and validation (B) datasets. (C and D) ROC curves of the diagnostic prediction models with the two DNA methylation-driven genes for training (C) and validation (D) datasets. (E and F) Unsupervised hierarchical clustering of two DNA methylation-driven genes for the diagnostic prediction model in training (E) and validation (F) datasets.

Adopting a logistic regression approach, we established a diagnostic model with two DNA methylation-driven genes to distinguish HCC from normal samples. Diagnostic scores were calculated using the following formula: logit (P = HCC) = 85.8918 - (2.8215 × SPP1 expression level) - (34.6788 × LCAT expression level). Applying the diagnostic model generated 100% specificity and 83.558% sensitivity for HCC in the training dataset (TCGA) of 50 normal samples and 371 HCC samples (Figure 8A) and 95.455% specificity and 95.111% sensitivity in the validation dataset (GSE14520) of 220 normal samples and 225 HCC samples (Figure 8B). We also showed that the model was capable of differentiating HCC from normal samples in both the training (AUC = 0.978) and validation (AUC = 0.981) datasets (Figures 8C and 8D). Unsupervised hierarchical clustering of these two DNA methylation-driven genes was capable of distinguishing HCC from normal samples with high sensitivity and specificity (Figures 8E and 8F).

**Figure 9:**
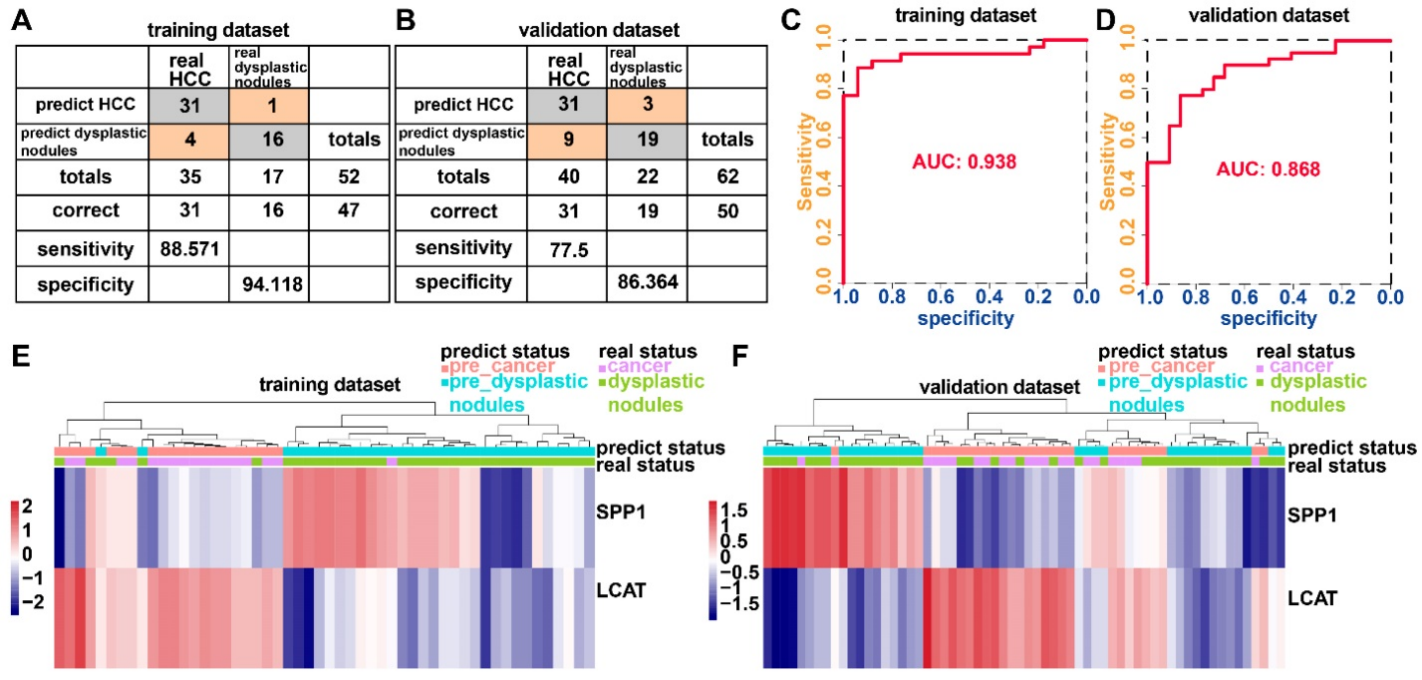
** Two DNA methylation-driven genes for distinguishing HCC from dysplastic nodules.** (A and B) Confusion matrices of binary results of the diagnostic prediction model for training (A) and validation (B) datasets. (C and D) ROC curves of the diagnostic prediction models with the two DNA methylation-driven genes for training (C) and validation (D) datasets. (E and F) Unsupervised hierarchical clustering of two DNA methylation-driven genes in the diagnostic prediction model for training (E) and validation (F) datasets.

The incidence of HCC is increasing in the United States and Europe 27. In 30-60% of cases in the West, early HCC diagnosis is feasible as a result of screening programs, enabling the application of curative treatments 27, 28. However, an increasing number of small nodules less than 2 cm are detected which are difficult to characterize by a pathological or radiological examination 29-31. We examined whether the diagnostic model based upon the two DNA methylation-driven genes can correctly distinguish between HCC and dysplastic nodules. Adopting a logistic regression approach, we established a diagnostic model with two DNA methylation-driven genes in the training cohort (GSE6764) of 52 samples (35 HCC and 17 dysplastic nodules), which was then externally validated in an independent dataset (GSE89377) of 62 samples (40 HCC and 22 dysplastic nodules). Diagnostic scores were calculated using the following formula: logit (P = HCC) = 22.9108 - (0.2558 × SPP1 expression level) - (2.5716 × LCAT expression level). The diagnostic model indicated an AUC of 0.938 for discriminating HCC from dysplastic nodules, with a sensitivity of 88.571% (31/35) and specificity of 94.118% (16/17) for the training cohort (Figures 9A and 9C). In the validation cohort, the diagnostic model yielded an AUC of 0.868, with a sensitivity of 77.5% (31/40) and specificity of 86.364% (19/22) (Figures 9B and 9D). Unsupervised hierarchical clustering of the diagnostic model was capable of distinguishing HCC from dysplastic nodules with high sensitivity and specificity (Figures 9 E and 9F).

### Validation of the expression pattern of DNA methylation-driven genes

**Figure 10:**
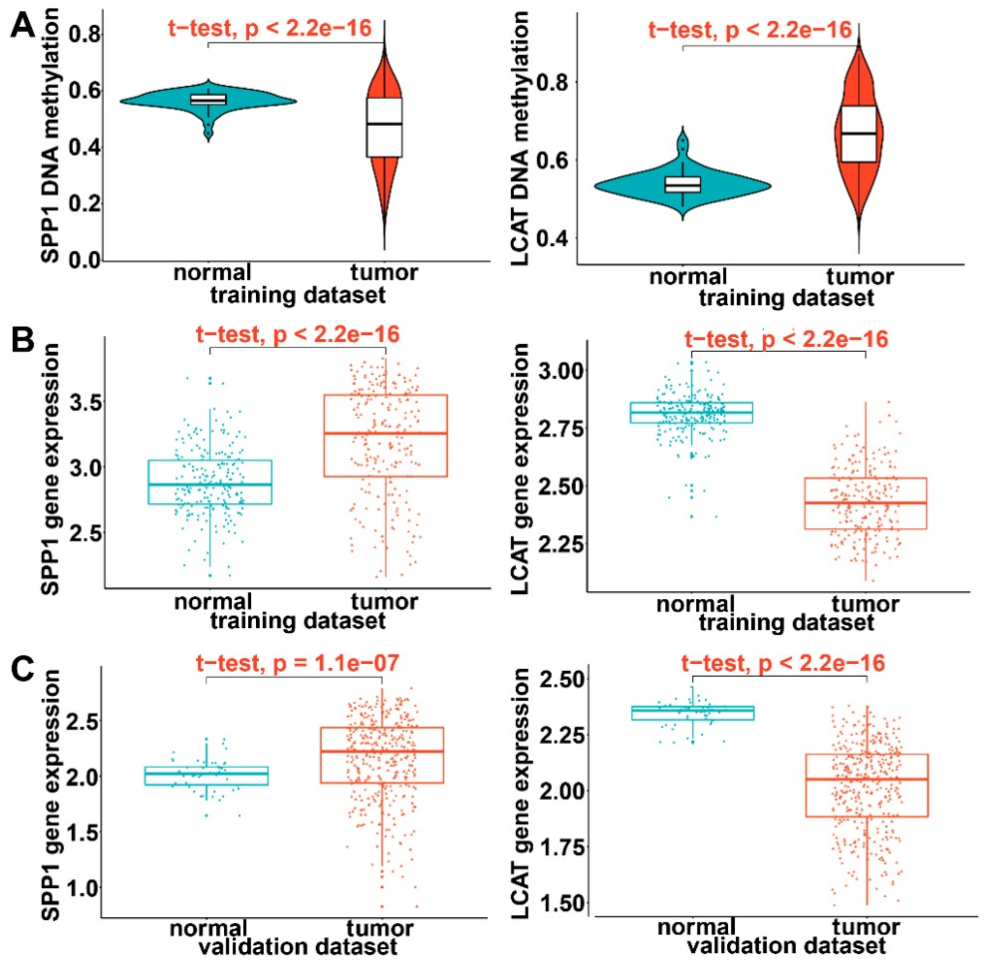
** Validation of expression of DNA methylation-driven genes.** (A) Violin plots of the DNA methylation status of two DNA methylation-driven genes in the training dataset. (B) Scatter plots of mRNA expression patterns of two DNA methylation-driven genes in the training dataset. (C) Scatter plots of mRNA expression patterns of two DNA methylation-driven genes in the validation dataset.

In the training dataset of HCC from TCGA, significantly low DNA methylation and high expression levels were noted for SPP1 and high DNA methylation and low expression for LCAT (P < 0.0001) (Figures 10A and 10B). This was consistent with prognostic and relapse analyses, demonstrating that SPP1 is a risk gene and LCAT a protective gene (Figures 10A and 10B). To further validate the expression levels of the two DNA methylation-driven genes in another database, these genes were selected from the GSE14520 validation dataset. As shown in Figure 10C, SPP1 exhibited significantly higher expression in tumor samples than in adjacent normal samples, whereas LCAT exhibited significantly decreased expression in tumor samples (P < 0.0001). In summary, these results demonstrated the expression levels of two DNA methylation-driven genes were useful for constructing the diagnostic, prognostic, and recurrence models.

### Validation of DNA methylation-driven genes in HCC

**Figure 11:**
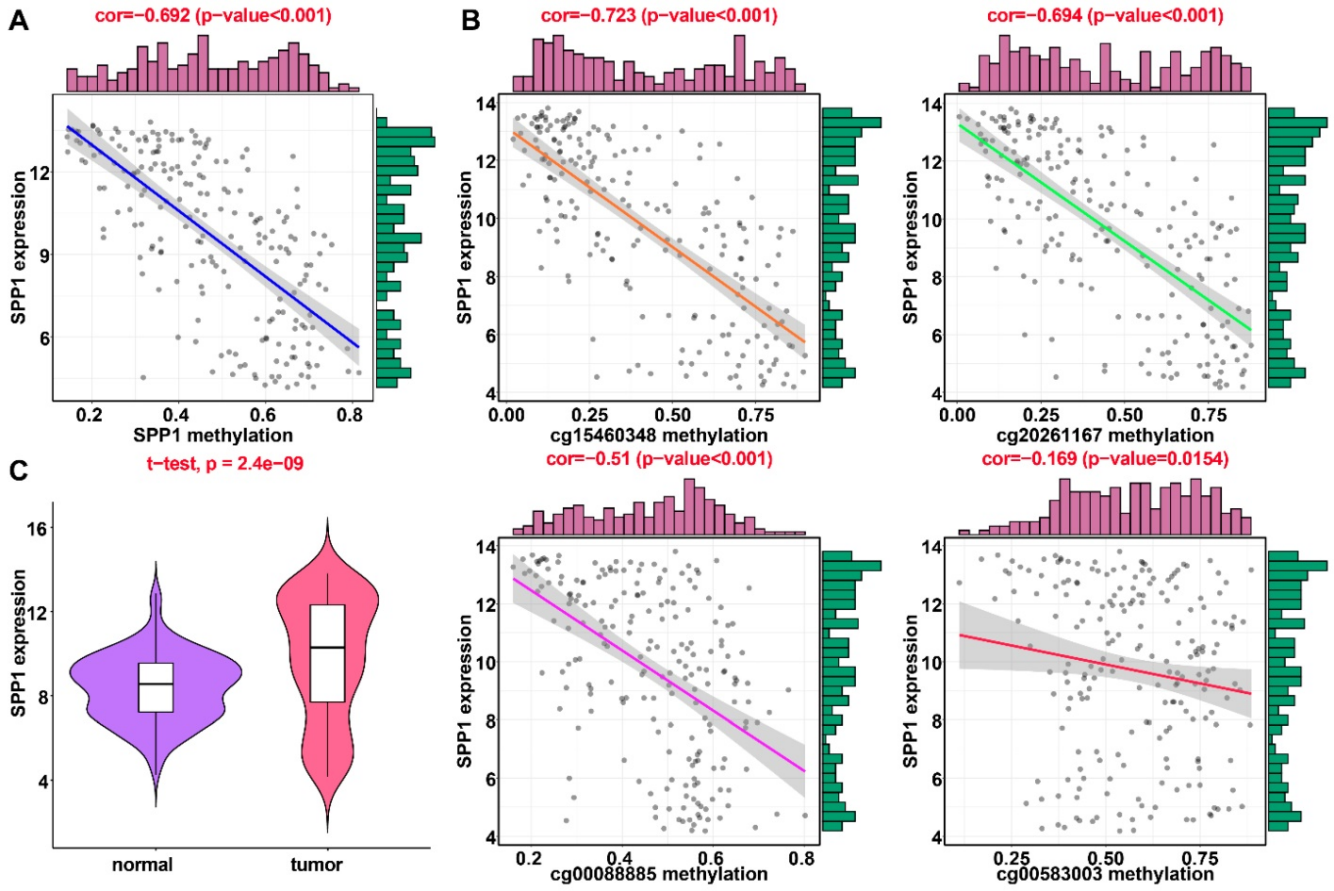
** Regression analysis between gene expression and DNA methylation of SPP1 in the GEO training dataset.** (A) Regression analysis between gene expression and DNA methylation of SPP1. (B) Regression analysis between gene expression and methylation of CpGs in the SPP1 promoter. The vertical axis represents DNA methylation of the DNA methylation-driven gene, and the horizontal axis denotes the expression of the DNA methylation-driven gene. The right and upper edges are histograms of DNA methylation and gene expression, respectively. (A) Violin plots of SPP1gene expression.

**Figure 12:**
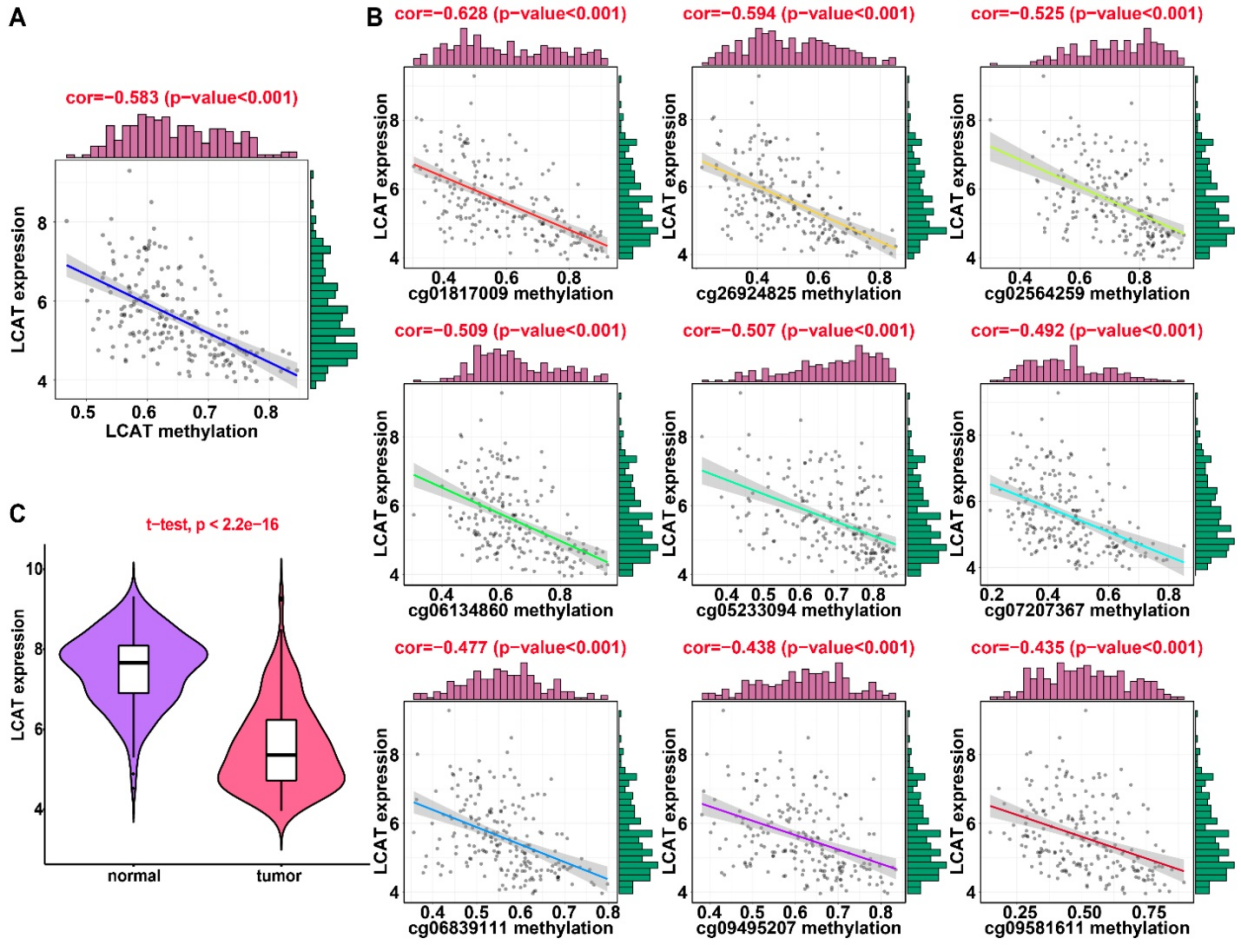
** Regression analysis between gene expression and DNA methylation of LCAT in the GEO training dataset.** (A) Regression analysis between gene expression and DNA methylation of LCAT. (B) Regression analysis between gene expression and DNA methylation of CpGs in the LCAT promoter. The vertical axis represents DNA methylation of the DNA methylation-driven gene, and the horizontal axis denotes expression of the DNA methylation-driven gene. The right and upper edges are histograms of DNA methylation and gene expression, respectively. (A) Violin plots of LCAT gene expression.

In the training dataset from TCGA, the average DNA methylation of CpGs in promoters and DNA methylation of all individual CpGs in the SPP1 promoter and 10 individual CpGs in the LCAT promoter were significantly negatively correlated with gene expression (Figures 1 and 2). To validate the regulatory relationships of DNA methylation-driven genes, an independent dataset containing a total of 214 HCC patients with gene expression profiling (GSE63898) and corresponding DNA methylation (GSE56588) downloaded from the GEO database was examined for further validation. Consistent with the results of the training dataset from TCGA, the average DNA methylation of CpGs in promoters and DNA methylation of all individual CpGs in the SPP1 promoter were significantly negatively correlated with gene expression (Figures 11A and 11B). SPP1 exhibited significantly higher expression in tumor samples than in corresponding normal samples (P < 0.0001) (Figure 11C). Also, the average DNA methylation of CpGs in promoters and DNA methylation of 9 of 10 (except cg00594148) individual CpGs in the LCAT promoter were significantly negatively correlated with gene expression (Figures 12A and 12B). LCAT exhibited significantly decreased expression in tumor tissues (P < 0.0001) (Figure 12C).

### Expression of SPP1 and LCAT in HepG2 cells after DAC treatment

As is evident from Figures 1C and 2C, cg15460348 and cg01817009 exhibited the strongest negative correlation with gene expression of SPP1 and LCAT, respectively (Figures 1C and 2C). We, therefore, analyzed changes in DNA methylation related to expression of SPP1 and LCAT after treatment of the HCC cell line HepG2 with the demethylation agent (DAC) to assess the functional relevance of SPP1 and LCAT DNA methylation in HCC. Our results indicated that DAC treatment reduced SPP1 and LCAT methylation and caused elevated SPP1 and LCAT expression in HepG2 cells (Figure S1).

## Discussion

As one of the most common malignant cancers, HCC is a public health burden 1, 32. Despite considerable progress in the treatment of early HCC, its 5-year survival rate has not improved significantly. Studies have shown that HCC, similar to other tumors, is caused by genetic changes as well as epigenetic abnormalities 5. Therefore, it is necessary to identify specific DNA methylation-affected genes and develop demethylation drugs with fewer adverse reactions, thus optimizing early HCC diagnosis, improving the prognosis of HCC, and enhancing HCC treatment.

The extensive use of high-throughput arrays has provided opportunities to find new genes involved in the epigenetic modulation of HCC 33. We attempted to further elucidate the function and significance of methylation in HCC by utilizing a comprehensive analytical instrument. Although high-throughput screening data from TCGA demonstrated the significant diversity of genetic alterations in HCC, not all identified abnormalities had a biological effect and facilitated HCC development 7, 34. For example, Fan et al. found no relationship by assessing the methylation status of promoters and RNA expression of 90 genes in 6 types of tissues 35.

When utilizing a high-throughput methodology with 450,000 probes, it is necessary to distinguish between epigenetic changes that promote a malignant phenotype and alterations of “passenger” genes without any biological effect. Therefore, we utilized a model-based instrument (MethylMix) to identify genes with aberrant methylation and linked the information to RNA-sequencing data that reflected gene expression 24. This integrative analysis has been performed for most cancers except for HCC, and its reliability has been demonstrated 36. Indeed, the combination of these complementary "omics" might help reveal clinically and biologically related information 37. In this research, we performed a multiomics HCC data analysis using MethylMix to identify DNA aberrant DNA methylation-driven genes that affect their expression. Subsequently, Cox proportional hazards regression analysis was conducted using expression data for DNA methylation-driven genes to generate a prognostic model. We found that the prediction models consisting of two DNA methylation-driven genes (SPP1 and LCAT) could be utilized as a prognostic factor for patients with HCC in TCGA and GEO. A nomogram comprising a prognostic model might help clinicians better manage patients with HCC. Furthermore, the recurrence and diagnostic models with these two genes were good at predicting HCC recurrence and diagnosis.

Previously, Baily et al. used 26 different bioinformatics tools to analyze the Multi-Center Mutation-Calling in Multiple Cancers (MC3) somatic mutation set and combined the results of manual curation to identify 299 cancer genes 38. More than 3400 predicted missense driver mutations supported by multiple lines of evidence were identified by sequence- and structure-based analyses. A total of 60-85% of putative mutations were confirmed as possible drivers by experimental validation. This discovery represents the most comprehensive endeavor to date to identify cancer driver genes and will be a critical reference for future clinical and biological efforts. However, the limitation of the gene list is its focus on small indels and point mutations without regard to other factors such as methylation events 38, as abnormal DNA methylation can also serve as a major driver of cancer 39. Global DNA methylation patterns are altered during tumorigenesis, causing hypermethylation of CpG islands and hypomethylation of non-CpG islands 40. In most types of cancers, DNA hypermethylation can cause deregulated silencing of several tumor suppressor genes (TSGs) 41, 42. DNA methylation, as an epigenetic process, can heritably alter gene expression without changing the DNA sequence 39.

In this study, our research focused on abnormally methylated genes driving tumor development and is a powerful complement to Baily and colleagues' research. Abnormal DNA methylation changes usually include two different states: hypermethylation and hypomethylation 8. Tumor progression can be accelerated when demethylation occurs at normal methylation sites. In this study, SPP1 was hypomethylated and expressed at a higher level in HCC than in nontumor tissues. SPP1, located at 4q22.1, is overexpressed in different malignant neoplasms including medullary thyroid cancer, colorectal cancer, and HCC and plays a role in metastasis and tumorigenesis 43-45. In colorectal cancer (CRC), the mRNA and protein expression of SPP1 are markedly higher in CRC tissues than in nontumor tissues 46. Overexpression of SPP1 is closely related to CRC metastasis, invasion, and poor survival 46. Furthermore, siRNA-SPP1 inhibits tumor growth, migration, proliferation, colony formation, and the cell cycle in vivo and enhances apoptosis in CRC cell lines 46. Also, protein expression of vimentin was downregulated and of E-cadherin apparently upregulated in CRC cells after siRNA-SPP1 treatment 46. SPP1 enhances CRC metastasis through activation of epithelial mesenchymal transition (EMT) 46. SPP1 was also found to be an aberrantly methylated hub gene that might participate in the progression and development of thyroid cancer (THCA) 47. SPP1 DNA methylation was significantly negatively correlated with SPP1 mRNA expression in THCA 47; it was hypomethylated and highly expressed in THCA 47.

Similarly, in our study, SPP1 was hypomethylated and highly expressed in HCC. Normally, SPP1 is expressed in stellate cells, Kupffer cells, and bile duct epithelium but is not expressed in liver cells 48. It has been reported that patients with HCC have higher serum SPP1 expression than those with chronic hepatitis, liver cirrhosis, or with normal livers 49, 50. Furthermore, extensive experimental and clinical evidence suggests that SPP1 is an attractive therapeutic target for the prevention of HCC metastasis and growth 51-57. SPP1 overexpression is related to early recurrence, intrahepatic metastasis, and unfavorable prognosis in HCC 58. Zhao et al. reported that SPP1 is overexpressed in HCC cell lines with higher metastatic potential and may regulate HCC growth by activating the MAPK pathway. Moreover, induction of MMP-2 production/activation and NF-kappa B (p65) translocation may be critical mechanisms underlying SPP1-mediated metastasis of HCC 51.

In summary, the in vivo and in vitro studies have demonstrated that SPP1 plays a critical role in the growth and metastasis of HCC. Zhao et al. found that silencing SPP1 leads to induction of mitochondria-mediated apoptosis, inhibits integrin expression, and blocks NF-κB activation, thereby suppressing the metastasis and growth of HCC 52. Thus, RNA interference-mediated depletion of SPP1 may be a promising strategy to treat HCC by sensitizing chemotherapeutic drugs. Yu et al. also found that SPP1 promotes HCC progression via PI3K/AKT/Twist signaling pathway 53. These data indicate that SPP1 is a driver gene that controls the growth and metastasis of HCC and is likely a promising target. Chen et al. established an independent prognostic signature (including gene KPNA2, CDC20, SPP1, and TOP2A) for patients with HCC, and Long et al. developed a four-gene-based model (HOXD9, MAGEB6, SPP1, and CENPA) that accurately predicted prognosis 45, 59. Deep mining of publicly available genomic data demonstrated that SPP1 is an important gene for HCC prognosis 45, 59. In our study, SPP1 was found to be a risk factor for HCC prognosis and recurrence, and the SPP1 models could accurately predict the OS and recurrence of HCC patients.

The CpG islands in the gene promoter regions are generally unmethylated under normal conditions 33. Methylation of CpG islands often leads to transcriptional gene silencing, which causes functional loss of significant genes, such as DNA repair genes and tumor suppressor genes, leading to abnormal growth regulation and differentiation of normal cells. Formation of various tumors is closely associated with whether the DNA damage can be repaired in a timely manner 33. Previous studies have demonstrated that LCAT gene expression was associated with DNA methylation. Zheng et al. established a four-gene-based prognostic model (SPINK1, TXNRD1, LCAT, and PZP) to predict OS in patients with HCC and found that the expression patterns of these four genes were closely associated with their methylation 60. Hlady et al. performed integrative analysis of multiple epigenetic modifications in HCC to identify epigenetic driver loci and demonstrated that 5mC progressively increased at the LCAT promoter during disease progression, with a corresponding decrease in its expression 61. In general, lower LCAT expression was related to poor prognosis 61. LCAT 5mC data from the TSS200 region was also significantly related to prognosis 61. In our study, LCAT was hypermethylated and downregulated in HCC compared with the nontumor tissue.

LCAT has been shown to generate cholesteryl esters (CEs) in the circulation of males from high-density lipoprotein (HDL) and transfer them to apolipoprotein (apo) B-containing lipoproteins with the help of lipid transfer protein (LTP) 62. LCAT is produced by the liver and secreted into the circulation and its activity can be attenuated in patients with liver disease 62. Additionally, the activity of plasma LCAT is reduced with HC damage progression, which is consistent with our data showing that LCAT is hypermethylated and minimally expressed in HCC. Given that methylation is potentially reversible, detection of aberrant oncogene and tumor suppressor gene DNA methylation in HCC might be useful for identifying therapeutic targets. These genes, especially SPP1 and LCAT, may become potential new molecular targets for the treatment of HCC, thereby preventing or even reversing the cancerization of cells by correcting abnormal DNA methylation.

In our study, a diagnostic model consisting of SPP1 and LCAT accurately diagnosed HCC, with AUCs reaching 0.978 in the training dataset and 0.981 in the validation dataset. Although our diagnostic model has not been evaluated using the sera of patients with HCC, the ability of serum detection of SPP1 and LCAT to diagnose HCC has been fully evaluated in previous studies 49. Shang et al. found that serum SPP1 was more sensitive than AFP for the diagnosis of HCC 49. Furthermore, a meta-analysis including 12 studies consisting of 1191 controls and 1235 patients with HCC concluded that the sensitivity of SPP1 was higher than that of AFP and that SPP1 was a comparable biomarker to AFP for the diagnosis of HCC and the combination of SPP1 and AFP could improve the sensitivity of early HCC diagnosis 63. Zhao et al. conducted an investigation to evaluate the possibility of LCAT as a marker for HCC, particularly as a biomarker in serum 64. Analysis of LCAT expression using qRT-PCR revealed that LCAT was expressed at lower levels in HCC specimens compared to adjacent normal tissues. At the protein level, LCAT showed a simultaneous reduction in HCC specimens as per Western blotting. In particular, LCAT could effectively distinguish between <2 cm HCC and healthy controls (AUC=0.9489). Furthermore, Western and dot-blot results showed a high correlation between LCAT expression in HCC tissues and matched serum samples. Although the diagnostic ability of LCAT has not been assessed in the sera of HCC patients, given the above results and the fact that it is a secretory protein, LCAT may serve as a promising noninvasive biomarker and improve the identification of HCC in patients with normal serum AFP.

Our study lays a foundation for the possibility of using SPP1 and LCAT as diagnostic biomarkers for HCC in serum samples of patients. In the future, we plan first to compare the expression of the candidate biomarkers and their concordance in the tissues and sera of HCC patients to validate the diagnostic ability of the potential serum biomarkers. Subsequently, we will develop a sensitive technique to detect the presence of SPP1 and LCAT in cell-free circulating tumor DNA (ctDNA), which will not only benefit patients who undergo surgery but will also help to screen patients with HCC.

Hepatocarcinogenesis is a multistep process that is characterized in most cirrhotic livers by progression from dysplastic nodules advancing to microscopic foci of HCC, which enlarge and replace the nodules developing into the initial stage of HCC and ultimately advanced HCC 65, 66. The systematic monitoring of cirrhotic patients by ultrasound aims to identify HCC at a very early stage (<2 cm) to ensure the highest probability of long-term survival. However, an increasing number of nodules are identified simultaneously that are difficult to characterize 29-31. Besides, morphological criteria for defining early stage HCC, such as stromal invasion, small cell dysplasia, CD34 expression, loss of reticulin, presence of pseudoglands, the thickness of liver cell plates, and cell density also exist in high-grade dysplastic nodules without a clear boundary between them 65. Therefore, morphological criteria for distinguishing dysplastic nodules and well-differentiated HCC are difficult to define, and strict lines between malignant and premalignant lesions cannot be drawn by simple microscopic observation even by expert pathologists 67. The inconsistency between the first pathological diagnosis and the final diagnosis obtained after the consensus of six pathologists was substantial, with moderate agreement using a weighted kappa coefficient 67. Thus, the identification of objective classifier genes or molecular biomarkers is eagerly anticipated and will assist in standardizing the histological differential diagnosis of these nodules critical for appropriate therapy. The novelty of our study resides in the diagnostic model that is effective in patients with small nodules and enables an objective, simple, and accurate diagnosis of HCC for routine clinical application.

In conclusion, four prediction models consisting of two DNA methylation-driven genes were developed and validated that have predictive value for HCC. Our findings support the notion that genes that are tightly controlled by DNA methylation are likely to be related to cancer outcomes. To the best of our knowledge, these are the first predictive models that employed DNA methylation-driven genes. Importantly, for the first time, only two genes were used to build diagnostic, prognostic, and recurrence models. In clinical practice, measuring the expression levels of only two genes is a cost-effective application and can provide accurate HCC diagnosis, prognosis, and predict recurrence. Although specifically developed for HCC, this proof of concept has broad utilization in tumors beyond HCC.

## Supplementary Material

Supplementary figures and tables.

## Abbreviations

HCC: Hepatocellular carcinoma
TCGA: The Cancer Genome Atlas
GEO: Gene Expression Omnibus
DEG: Differentially expressed gene
FC: Fold change
FDR: False discovery rate
LASSO: Least absolute shrinkage and selection operator
SOCS1: Suppressor of cytokine signaling 1
ROC: Receiver operating characteristic
AFP: Alpha-fetoprotein
CRC: Colorectal cancer
C-index: Concordance index
OS: Overall survival
SPP1: Secreted phosphoprotein 1
LCAT: Lecithin-cholesterol acyltransferase
AUC: Area under the curve
CI: Confidence interval
HR: Hazard ratio
EMT: Epithelial-mesenchymal transition
THCA: Thyroid cancer
LTP: Lipid transfer protein
CEs: Cholesteryl esters
HDL: High-density lipoprotein
MC3: Multi-Center Mutation-Calling in Multiple Cancers
TSGs: Tumor suppressor genes
ctDNA: Circulating tumor DNA
