# Supplementary material for: DNA methylation-driven genes for constructing diagnostic, prognostic, and recurrence models for hepatocellular carcinoma
Source: Theranostics. 2019 Sep 25;9(24):7251–67. doi: 10.7150/thno.31155 (PMC6831284; doi:10.7150/thno.31155)
Supplement: Supplementary file 1 — Supplementary figures and tables. [file thnov09p7251s1.pdf]

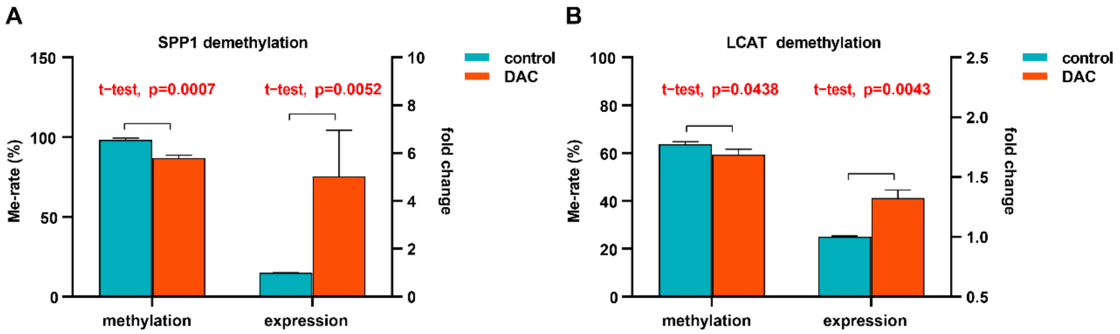

Figure S1. Demethylation of SPP1 and LCAT by DAC.

Levels of SPP1 (A) and LCAT (B) methylation and expression after demethylation.

Table S1. The accession number of the sample set from TCGA.

**The accession number of RNA-sequencing profiles of 371 HCC samples**	TCGA-2Y-A9H4-01A	TCGA-DD-AAW0-01A
	TCGA-PD-A5DF-01A	TCGA-G3-A5SK-01A
	TCGA-2Y-A9H7-01A	TCGA-DD-A4NK-01A
	TCGA-2Y-A9H0-01A	TCGA-BC-A10Y-01A
	TCGA-RC-A7SF-01A	TCGA-BC-A5W4-01A
	TCGA-EP-A12J-01A	TCGA-BC-A8YO-01A
	TCGA-DD-A39V-01A	TCGA-DD-A1EH-01A
	TCGA-DD-AAD0-01A	TCGA-DD-A4NH-01A
	TCGA-DD-AADF-01A	TCGA-2Y-A9GZ-01A
	TCGA-FV-A4ZQ-01A	TCGA-BD-A2L6-01A
	TCGA-DD-AADB-01A	TCGA-2Y-A9H8-01A
	TCGA-KR-A7K8-01A	TCGA-2V-A95S-01A
	TCGA-EP-A3JL-01A	TCGA-DD-A4NQ-01A
	TCGA-BC-A69I-01A	TCGA-CC-A9FW-01A
	TCGA-BC-A217-01A	TCGA-DD-AADS-01A
	TCGA-ED-A8O5-01A	TCGA-UB-A7ME-01A
	TCGA-K7-AAU7-01A	TCGA-CC-A9FS-01A
	TCGA-ZP-A9D1-01A	TCGA-UB-A7MA-01A
	TCGA-FV-A2QR-01A	TCGA-BW-A5NO-01A
	TCGA-DD-A73F-01A	TCGA-DD-A73C-01A
	TCGA-ED-A97K-01A	TCGA-DD-A4NJ-01A
	TCGA-BC-A10Z-01A	TCGA-DD-A4NI-01A
	TCGA-G3-A25V-01A	TCGA-G3-A5SI-01A
	TCGA-CC-A7II-01A	TCGA-G3-A5SM-01A
	TCGA-DD-A3A6-01A	TCGA-G3-AAV6-01A
	TCGA-DD-AAW2-01A	TCGA-DD-A1EC-01A
	TCGA-DD-AACI-01A	TCGA-BC-A216-01A
	TCGA-DD-A116-01A	TCGA-DD-AACE-01A
	TCGA-DD-AAVZ-01A	TCGA-BC-A10S-01A
	TCGA-RC-A6M3-01A	TCGA-CC-A3MC-01A
	TCGA-DD-AADL-01A	TCGA-DD-AACS-01A

	TCGA-BD-A3ER-01A	TCGA-NI-A8LF-01A
	TCGA-CC-A1HT-01A	TCGA-UB-A7MB-01A
	TCGA-ED-A66X-01A	TCGA-CC-A7IG-01A
	TCGA-XR-A8TG-01A	TCGA-2Y-A9H3-01A
	TCGA-G3-AAV4-01A	TCGA-2Y-A9H5-01A
	TCGA-DD-A4NR-01A	TCGA-G3-A7M9-01A
	TCGA-DD-A3A9-01A	TCGA-BC-A110-01A
	TCGA-BC-A112-01A	TCGA-MI-A75H-01A
	TCGA-WX-AA47-01A	TCGA-DD-AAVW-01A
	TCGA-CC-A5UD-01A	TCGA-ED-A7PZ-01A
	TCGA-DD-AAE4-01A	TCGA-BC-A10U-01A
	TCGA-RG-A7D4-01A	TCGA-5R-AA1D-01A
	TCGA-DD-A4NP-01A	TCGA-DD-AACF-01A
	TCGA-ED-A82E-01A	TCGA-ZS-A9CG-01A
	TCGA-DD-AAEE-01A	TCGA-G3-A7M5-01A
	TCGA-UB-AA0U-01A	TCGA-G3-A7M7-01A
	TCGA-RC-A7SH-01A	TCGA-CC-A3MA-01A
	TCGA-CC-A7IL-01A	TCGA-DD-AACU-01A
	TCGA-G3-A3CI-01A	TCGA-UB-A7MC-01A
	TCGA-DD-AAEB-01A	TCGA-DD-AAEH-01A
	TCGA-EP-A26S-01A	TCGA-DD-A3A4-01A
	TCGA-CC-5261-01A	TCGA-BW-A5NP-01A
	TCGA-CC-A8HS-01A	TCGA-DD-AAEK-01A
	TCGA-DD-A4NO-01A	TCGA-EP-A2KA-01A
	TCGA-DD-A11A-01A	TCGA-EP-A3RK-01A
	TCGA-ED-A7PY-01A	TCGA-G3-A3CH-01A
	TCGA-DD-AAVR-01A	TCGA-DD-AADK-01A
	TCGA-DD-AAD5-01A	TCGA-DD-A4NA-01A
	TCGA-DD-AAE3-01A	TCGA-CC-5263-01A
	TCGA-DD-A4NN-01A	TCGA-5C-A9VG-01A
	TCGA-RC-A7SK-01A	TCGA-2Y-A9GV-01A
	TCGA-G3-A5SL-01A	TCGA-ZP-A9CY-01A
	TCGA-DD-AAW3-01A	TCGA-DD-A73A-01A
	TCGA-DD-A4NS-01A	TCGA-DD-AAD3-01A
	TCGA-DD-A39X-01A	TCGA-WQ-A9G7-01A
	TCGA-CC-5259-01A	TCGA-CC-A123-01A
	TCGA-ED-A5KG-01A	TCGA-WQ-AB4B-01A
	TCGA-GJ-A6C0-01A	TCGA-ES-A2HS-01A
	TCGA-DD-A3A5-01A	TCGA-CC-A7IF-01A
	TCGA-BC-A10Q-01A	TCGA-ZP-A9D2-01A
	TCGA-DD-AACN-01A	TCGA-ZS-A9CD-01A
	TCGA-DD-AAE6-01A	TCGA-DD-AAD1-01A
	TCGA-2Y-A9H6-01A	TCGA-DD-A3A2-01A

	TCGA-DD-AADG-01A	TCGA-HP-A5MZ-01A
	TCGA-FV-A4ZP-01A	TCGA-G3-A25Y-01A
	TCGA-G3-A25T-01A	TCGA-DD-A4NV-01A
	TCGA-DD-AAVS-01A	TCGA-5C-A9VH-01A
	TCGA-DD-AACQ-01A	TCGA-KR-A7K2-01A
	TCGA-DD-A11C-01A	TCGA-EP-A2KC-01A
	TCGA-DD-AADC-01A	TCGA-G3-AAUZ-01A
	TCGA-DD-A39Y-01A	TCGA-DD-A1EJ-01A
	TCGA-DD-AA3A-01A	TCGA-RC-A7S9-01A
	TCGA-CC-A7IE-01A	TCGA-DD-AAC8-01A
	TCGA-EP-A2KB-01A	TCGA-ZS-A9CE-01A
	TCGA-DD-A4NE-01A	TCGA-K7-A5RF-01A
	TCGA-O8-A75V-01A	TCGA-DD-AADO-01A
	TCGA-ZP-A9D0-01A	TCGA-DD-A39W-01A
	TCGA-DD-A11B-01A	TCGA-G3-AAV0-01A
	TCGA-G3-A6UC-01A	TCGA-DD-A73D-01A
	TCGA-CC-A3M9-01A	TCGA-DD-A11D-01A
	TCGA-2Y-A9H2-01A	TCGA-DD-AACJ-01A
	TCGA-XR-A8TF-01A	TCGA-ES-A2HT-01A
	TCGA-DD-AACX-01A	TCGA-CC-A3MB-01A
	TCGA-G3-AAV1-01A	TCGA-CC-5258-01A
	TCGA-ZP-A9D4-01A	TCGA-HP-A5N0-01A
	TCGA-ED-A66Y-01A	TCGA-WX-AA46-01A
	TCGA-NI-A4U2-01A	TCGA-DD-A4NB-01A
	TCGA-XR-A8TC-01A	TCGA-DD-AADM-01A
	TCGA-DD-AACC-01A	TCGA-DD-AACZ-01A
	TCGA-G3-A25S-01A	TCGA-DD-AAW1-01A
	TCGA-QA-A7B7-01A	TCGA-2Y-A9H1-01A
	TCGA-DD-AACW-01A	TCGA-ED-A7PX-01A
	TCGA-G3-A7M8-01A	TCGA-DD-A118-01A
	TCGA-DD-A73B-01A	TCGA-G3-A3CG-01A
	TCGA-CC-A7IK-01A	TCGA-DD-AADN-01A
	TCGA-DD-AAVX-01A	TCGA-DD-AAVP-01A
	TCGA-DD-AAEI-01A	TCGA-DD-AAEG-01A
	TCGA-BC-A3KG-01A	TCGA-G3-A3CK-01A
	TCGA-K7-A6G5-01A	TCGA-DD-AACG-01A
	TCGA-G3-A25Z-01A	TCGA-ZS-A9CF-01A
	TCGA-DD-AAE1-01A	TCGA-BC-4073-01B
	TCGA-DD-AACB-01A	TCGA-DD-AADQ-01A
	TCGA-LG-A9QC-01A	TCGA-DD-AAD8-01A
	TCGA-2Y-A9GU-01A	TCGA-DD-AAVY-01A
	TCGA-RC-A6M5-01A	TCGA-MI-A75G-01A
	TCGA-DD-A3A3-01A	TCGA-DD-A4ND-01A

	TCGA-DD-A73G-01A	TCGA-MI-A75E-01A
	TCGA-DD-A114-01A	TCGA-DD-A39Z-01A
	TCGA-ED-A8O6-01A	TCGA-RC-A6M4-01A
	TCGA-G3-A3CJ-01A	TCGA-DD-AACP-01A
	TCGA-CC-5262-01A	TCGA-XR-A8TE-01A
	TCGA-CC-A5UE-01A	TCGA-DD-AADR-01A
	TCGA-DD-A4NF-01A	TCGA-G3-A7M6-01A
	TCGA-2Y-A9GY-01A	TCGA-DD-AAD6-01A
	TCGA-LG-A9QD-01A	TCGA-DD-AAED-01A
	TCGA-FV-A23B-01A	TCGA-DD-AADW-01A
	TCGA-DD-A1EF-01A	TCGA-FV-A495-01A
	TCGA-DD-A4NG-01A	TCGA-DD-AAC9-01A
	TCGA-DD-AADP-01A	TCGA-DD-AADY-01A
	TCGA-FV-A3R3-01A	TCGA-DD-AAEA-01A
	TCGA-G3-AAV3-01A	TCGA-BC-A3KF-01A
	TCGA-BD-A3EP-01A	TCGA-CC-A5UC-01A
	TCGA-MI-A75I-01A	TCGA-CC-A8HV-01A
	TCGA-DD-A1EL-01A	TCGA-BC-A10X-01A
	TCGA-2Y-A9GX-01A	TCGA-WX-AA44-01A
	TCGA-DD-A4NL-01A	TCGA-KR-A7K0-01A
	TCGA-BW-A5NQ-01A	TCGA-DD-AACL-01A
	TCGA-CC-5260-01A	TCGA-ED-A627-01A
	TCGA-ED-A7XO-01A	TCGA-ED-A459-01A
	TCGA-2Y-A9GS-01A	TCGA-DD-AACO-01A
	TCGA-DD-A1EE-01A	TCGA-DD-AACV-01A
	TCGA-DD-A3A7-01A	TCGA-DD-A1EK-01A
	TCGA-DD-AADA-01A	TCGA-FV-A3I0-01A
	TCGA-DD-AACD-01A	TCGA-G3-AAV7-01A
	TCGA-MI-A75C-01A	TCGA-DD-AAE2-01A
	TCGA-FV-A2QQ-01A	TCGA-ZP-A9CZ-01A
	TCGA-BC-A10W-01A	TCGA-DD-A3A8-01A
	TCGA-BC-A10R-01A	TCGA-BC-A69H-01A
	TCGA-ZP-A9CV-01A	TCGA-G3-AAV5-01A
	TCGA-DD-AACT-01A	TCGA-CC-A8HU-01A
	TCGA-DD-A1ED-01A	TCGA-ED-A4XI-01A
	TCGA-DD-AAVQ-01A	TCGA-CC-A9FV-01A
	TCGA-2Y-A9H9-01A	TCGA-CC-A8HT-01A
	TCGA-4R-AA8I-01A	TCGA-KR-A7K7-01A
	TCGA-UB-AA0V-01A	TCGA-DD-A1EG-01A
	TCGA-3K-AAZ8-01A	TCGA-GJ-A3OU-01A
	TCGA-DD-AACA-01A	TCGA-DD-AAE9-01A
	TCGA-DD-AAVU-01A	TCGA-DD-A1EA-01A
	TCGA-MR-A8JO-01A	TCGA-FV-A3I1-01A

	TCGA-BC-A10T-01A	TCGA-YA-A8S7-01A
	TCGA-DD-AAD2-01A	TCGA-DD-AADD-01A
	TCGA-UB-A7MF-01A	TCGA-DD-AAVV-01A
	TCGA-CC-A7IH-01A	TCGA-G3-AAV2-01A
	TCGA-DD-AADI-01A	TCGA-G3-A25X-01A
	TCGA-RC-A6M6-01A	TCGA-CC-A9FU-01A
	TCGA-RC-A7SB-01A	TCGA-DD-A1EI-01A
	TCGA-DD-A3A1-01A	TCGA-DD-A73E-01A
	TCGA-5R-AA1C-01A	TCGA-T1-A6J8-01A
	TCGA-ED-A7XP-01A	TCGA-MR-A520-01A
	TCGA-2Y-A9HB-01A	TCGA-2Y-A9GW-01A
	TCGA-XR-A8TD-01A	TCGA-G3-A5SJ-01A
	TCGA-5C-AAPD-01A	TCGA-DD-AADJ-01A
	TCGA-LG-A6GG-01A	TCGA-DD-AAE0-01A
	TCGA-FV-A3R2-01A	TCGA-FV-A496-01A
	TCGA-2Y-A9GT-01A	TCGA-DD-AAE7-01A
	TCGA-DD-A1EB-01A	TCGA-CC-A7IJ-01A
	TCGA-GJ-A9DB-01A	TCGA-DD-AADV-01A
	TCGA-K7-A5RG-01A	TCGA-DD-AACY-01A
	TCGA-2Y-A9HA-01A	TCGA-DD-AACH-01A
	TCGA-5R-AAAM-01A	TCGA-DD-A113-01A
	TCGA-G3-A25U-01A	TCGA-DD-A115-01A
	TCGA-BC-4072-01B	TCGA-DD-AADU-01A
	TCGA-CC-5264-01A	TCGA-DD-AACK-01A
	TCGA-UB-A7MD-01A	TCGA-WJ-A86L-01A
	TCGA-DD-A119-01A	
**The accession number of RNA-sequencing profiles of 50 adjacent nontumor samples**	TCGA-FV-A23B-11A	TCGA-DD-A39V-11A
	TCGA-BC-A216-11A	TCGA-DD-A3A3-11A
	TCGA-BC-A10T-11A	TCGA-DD-A1EL-11A
	TCGA-BC-A10U-11A	TCGA-FV-A3R2-11A
	TCGA-DD-A3A5-11A	TCGA-DD-A1EI-11A
	TCGA-G3-A3CH-11A	TCGA-BC-A110-11A
	TCGA-DD-A39X-11A	TCGA-BC-A10R-11A
	TCGA-DD-A1EC-11A	TCGA-DD-A1EE-11A
	TCGA-EP-A26S-11A	TCGA-DD-A118-11A
	TCGA-DD-A39Z-11A	TCGA-FV-A2QR-11A
	TCGA-BC-A10W-11A	TCGA-DD-A3A1-11A
	TCGA-BC-A10X-11A	TCGA-DD-A3A6-11A
	TCGA-BC-A10Z-11A	TCGA-DD-A3A8-11A
	TCGA-DD-A3A4-11A	TCGA-DD-A39W-11A
	TCGA-DD-A119-11A	TCGA-DD-A11A-11A
	TCGA-DD-A116-11A	TCGA-DD-A11D-11A
	TCGA-DD-A11C-11A	TCGA-DD-A11B-11A

	TCGA-ES-A2HT-11A	TCGA-DD-A114-11A
	TCGA-EP-A12J-11A	TCGA-BD-A3EP-11A
	TCGA-FV-A3I0-11A	TCGA-DD-A3A2-11A
	TCGA-EP-A3RK-11A	TCGA-DD-A113-11A
	TCGA-BD-A2L6-11A	TCGA-DD-A1EB-11A
	TCGA-DD-A1EH-11A	TCGA-BC-A10Y-11A
	TCGA-DD-A1EG-11A	TCGA-DD-A1EJ-11A
	TCGA-FV-A3I1-11A	TCGA-BC-A10Q-11A
**The accession number of DNA methylation profiles of 380 HCC samples**	TCGA-ZP-A9D0-01A	TCGA-DD-AACG-01A
	TCGA-DD-A1EJ-01A	TCGA-HP-A5MZ-01A
	TCGA-CC-A8HT-01A	TCGA-BC-A3KF-01A
	TCGA-WX-AA44-01A	TCGA-DD-AAD2-01A
	TCGA-FV-A496-01A	TCGA-O8-A75V-01A
	TCGA-4R-AA8I-01A	TCGA-G3-A7M5-01A
	TCGA-G3-A5SJ-01A	TCGA-ZS-A9CD-01A
	TCGA-DD-AAD3-01A	TCGA-DD-AAE8-01A
	TCGA-EP-A3JL-01A	TCGA-ED-A97K-01A
	TCGA-DD-A3A9-01A	TCGA-G3-A7M9-01A
	TCGA-CC-A8HU-01A	TCGA-DD-AAW0-01A
	TCGA-2Y-A9H7-01A	TCGA-BC-4073-01B
	TCGA-K7-A5RG-01A	TCGA-DD-AACY-01A
	TCGA-CC-A7IE-01A	TCGA-DD-AADF-01A
	TCGA-DD-AADI-01A	TCGA-XR-A8TD-01A
	TCGA-G3-A5SK-01A	TCGA-G3-A25S-01A
	TCGA-NI-A4U2-01A	TCGA-MI-A75C-01A
	TCGA-CC-A7IG-01A	TCGA-BC-A112-01A
	TCGA-DD-AADC-01A	TCGA-G3-AAV5-01A
	TCGA-DD-A4NB-01A	TCGA-DD-A1EH-01A
	TCGA-DD-A4NE-01A	TCGA-DD-A1EA-01A
	TCGA-DD-AACM-01A	TCGA-DD-A73F-01A
	TCGA-DD-A1EL-01A	TCGA-CC-A123-01A
	TCGA-DD-AADW-01A	TCGA-EP-A26S-01A
	TCGA-CC-A9FW-01A	TCGA-DD-A39Z-01A
	TCGA-DD-AAE6-01A	TCGA-5C-AAPD-01A
	TCGA-WQ-AB4B-01A	TCGA-DD-AAVU-01A
	TCGA-CC-A3M9-01A	TCGA-CC-A5UC-01A
	TCGA-BC-A5W4-01A	TCGA-G3-A5SL-01A
	TCGA-DD-AAW1-01A	TCGA-EP-A2KA-01A
	TCGA-BC-A69H-01A	TCGA-2Y-A9GY-01A
	TCGA-DD-AACQ-01A	TCGA-ED-A66X-01A
	TCGA-G3-AAV2-01A	TCGA-ES-A2HT-01A
	TCGA-DD-A4NS-01A	TCGA-2Y-A9H1-01A
	TCGA-DD-AAD0-01A	TCGA-WQ-A9G7-01A

	TCGA-G3-AAV7-01A	TCGA-DD-A3A7-01A
	TCGA-BC-4072-01B	TCGA-DD-AAW3-01A
	TCGA-2Y-A9H8-01A	TCGA-DD-AADD-01A
	TCGA-2Y-A9GW-01A	TCGA-DD-A73B-01A
	TCGA-UB-AA0U-01A	TCGA-DD-AAEA-01A
	TCGA-G3-A3CK-01A	TCGA-DD-A1EE-01A
	TCGA-CC-A9FU-01A	TCGA-G3-AAV6-01A
	TCGA-DD-A4NV-01A	TCGA-EP-A3RK-01A
	TCGA-G3-AAV4-01A	TCGA-DD-AACF-01A
	TCGA-DD-AAVS-01A	TCGA-FV-A3R3-01A
	TCGA-CC-A7IJ-01A	TCGA-G3-A25U-01A
	TCGA-DD-AADS-01A	TCGA-DD-AAVW-01A
	TCGA-DD-AACA-02B	TCGA-ED-A7XP-01A
	TCGA-DD-A4NJ-01A	TCGA-K7-A5RF-01A
	TCGA-G3-A25T-01A	TCGA-DD-A3A3-01A
	TCGA-DD-A11B-01A	TCGA-HP-A5N0-01A
	TCGA-BC-A216-01A	TCGA-DD-AACA-02A
	TCGA-ED-A66Y-01A	TCGA-2Y-A9HB-01A
	TCGA-DD-AADJ-01A	TCGA-DD-A3A6-01A
	TCGA-RC-A6M5-01A	TCGA-PD-A5DF-01A
	TCGA-DD-A3A5-01A	TCGA-BD-A2L6-01A
	TCGA-DD-AADY-01A	TCGA-BC-A10Q-01A
	TCGA-G3-AAV3-01A	TCGA-5R-AAAM-01A
	TCGA-YA-A8S7-01A	TCGA-DD-A1EB-01A
	TCGA-ZP-A9D1-01A	TCGA-DD-AACP-01A
	TCGA-DD-AACV-01A	TCGA-DD-AADM-01A
	TCGA-DD-A3A4-01A	TCGA-ZS-A9CG-01A
	TCGA-DD-AAVX-01A	TCGA-DD-AADA-01A
	TCGA-DD-AAE7-01A	TCGA-CC-A1HT-01A
	TCGA-CC-A5UE-01A	TCGA-FV-A3I0-01A
	TCGA-ED-A7PZ-01A	TCGA-ED-A7PY-01A
	TCGA-DD-AAEB-01A	TCGA-RC-A6M3-01A
	TCGA-DD-AACI-01A	TCGA-2Y-A9H9-01A
	TCGA-DD-AACE-01A	TCGA-ES-A2HS-01A
	TCGA-2Y-A9GZ-01A	TCGA-DD-AACC-01A
	TCGA-DD-A4NO-01A	TCGA-DD-AAD5-01A
	TCGA-DD-A73E-01A	TCGA-DD-A1EK-01A
	TCGA-BC-A10T-01A	TCGA-DD-AAEE-01A
	TCGA-2Y-A9GX-01A	TCGA-K7-AAU7-01A
	TCGA-CC-A7IH-01A	TCGA-BC-A10W-01A
	TCGA-FV-A2QR-01A	TCGA-MI-A75G-01A
	TCGA-CC-A3MC-01A	TCGA-DD-AAE1-01A
	TCGA-G3-A5SM-01A	TCGA-DD-A119-01A

	TCGA-DD-AAVP-01A	TCGA-QA-A7B7-01A
	TCGA-DD-AAEH-01A	TCGA-DD-A4NI-01A
	TCGA-G3-AAV0-01A	TCGA-DD-A1EI-01A
	TCGA-ZP-A9CZ-01A	TCGA-DD-A73G-01A
	TCGA-DD-A4NF-01A	TCGA-K7-A6G5-01A
	TCGA-RG-A7D4-01A	TCGA-DD-AACA-01A
	TCGA-DD-AACL-01A	TCGA-CC-A8HV-01A
	TCGA-2V-A95S-01A	TCGA-CC-A7IK-01A
	TCGA-DD-A114-01A	TCGA-5C-A9VH-01A
	TCGA-DD-A116-01A	TCGA-LG-A9QC-01A
	TCGA-DD-A4NL-01A	TCGA-DD-A4NH-01A
	TCGA-DD-AAC8-01A	TCGA-XR-A8TC-01A
	TCGA-2Y-A9GU-01A	TCGA-DD-AACK-01A
	TCGA-CC-A9FS-01A	TCGA-MR-A8JO-01A
	TCGA-ZP-A9D4-01A	TCGA-EP-A2KC-01A
	TCGA-DD-AAEI-01A	TCGA-DD-A115-01A
	TCGA-DD-A4NG-01A	TCGA-DD-AACN-01A
	TCGA-DD-A3A2-01A	TCGA-ZS-A9CF-01A
	TCGA-DD-A4NN-01A	TCGA-UB-A7MD-01A
	TCGA-DD-AAE0-01A	TCGA-DD-A39X-01A
	TCGA-5R-AA1D-01A	TCGA-G3-A3CJ-01A
	TCGA-UB-AA0V-01A	TCGA-CC-5262-01A
	TCGA-ED-A82E-01A	TCGA-DD-AACS-01A
	TCGA-DD-A39V-01A	TCGA-WJ-A86L-01A
	TCGA-DD-AADK-01A	TCGA-DD-AAE2-01A
	TCGA-G3-A7M8-01A	TCGA-DD-A1ED-01A
	TCGA-G3-A3CH-01A	TCGA-DD-A73C-01A
	TCGA-RC-A7SH-01A	TCGA-EP-A12J-01A
	TCGA-G3-A7M6-01A	TCGA-DD-AAD8-01A
	TCGA-ED-A627-01A	TCGA-CC-5264-01A
	TCGA-ZP-A9D2-01A	TCGA-DD-A1EG-01A
	TCGA-G3-A25W-01A	TCGA-DD-A1E9-01A
	TCGA-GJ-A3OU-01A	TCGA-BD-A3EP-01A
	TCGA-T1-A6J8-01A	TCGA-ZS-A9CF-02A
	TCGA-GJ-A9DB-01A	TCGA-BC-A10Z-01A
	TCGA-MI-A75E-01A	TCGA-ED-A7PX-01A
	TCGA-BC-A3KG-01A	TCGA-DD-AADP-01A
	TCGA-CC-A7IF-01A	TCGA-EP-A2KB-01A
	TCGA-DD-AAE9-01A	TCGA-2Y-A9HA-01A
	TCGA-UB-A7MB-01A	TCGA-BC-A10U-01A
	TCGA-ED-A8O6-01A	TCGA-DD-A4NP-01A
	TCGA-DD-A39W-01A	TCGA-DD-AAVZ-01A
	TCGA-KR-A7K7-01A	TCGA-BC-A110-01A

	TCGA-G3-A6UC-01A	TCGA-RC-A6M6-01A
	TCGA-G3-A25Y-01A	TCGA-DD-A3A8-01A
	TCGA-DD-AAED-01A	TCGA-CC-5258-01A
	TCGA-DD-A4NA-01A	TCGA-XR-A8TE-01A
	TCGA-UB-A7MF-01A	TCGA-DD-A113-01A
	TCGA-ED-A5KG-01A	TCGA-FV-A3R2-01A
	TCGA-DD-AACD-01A	TCGA-CC-A7II-01A
	TCGA-DD-AAVR-01A	TCGA-FV-A23B-01A
	TCGA-CC-A7IL-01A	TCGA-RC-A7SB-01A
	TCGA-2Y-A9H3-01A	TCGA-CC-5261-01A
	TCGA-UB-A7ME-01A	TCGA-DD-AADR-01A
	TCGA-DD-AACH-01A	TCGA-DD-A4ND-01A
	TCGA-5R-AA1C-01A	TCGA-CC-A5UD-01A
	TCGA-DD-A3A1-01A	TCGA-DD-A39Y-01A
	TCGA-ED-A4XI-01A	TCGA-G3-A5SI-01A
	TCGA-DD-A11A-01A	TCGA-KR-A7K8-01A
	TCGA-ED-A7XO-01A	TCGA-2Y-A9H2-01A
	TCGA-CC-A8HS-01A	TCGA-WX-AA46-01A
	TCGA-LG-A6GG-01A	TCGA-2Y-A9GT-01A
	TCGA-CC-A3MA-01A	TCGA-DD-AACX-01A
	TCGA-CC-5263-01A	TCGA-DD-AADN-01A
	TCGA-BW-A5NP-01A	TCGA-ED-A459-01A
	TCGA-FV-A4ZQ-01A	TCGA-DD-A11D-01A
	TCGA-ZS-A9CE-01A	TCGA-DD-AADQ-01A
	TCGA-DD-A1EC-01A	TCGA-BC-A217-01A
	TCGA-ZP-A9CY-01A	TCGA-CC-5259-01A
	TCGA-GJ-A6C0-01A	TCGA-2Y-A9H4-01A
	TCGA-DD-A4NQ-01A	TCGA-KR-A7K2-01A
	TCGA-DD-AACU-01A	TCGA-3K-AAZ8-01A
	TCGA-WX-AA47-01A	TCGA-DD-AA3A-01A
	TCGA-2Y-A9GV-01A	TCGA-FV-A4ZP-01A
	TCGA-G3-AAV1-01A	TCGA-2Y-A9H5-01A
	TCGA-DD-A4NK-01A	TCGA-DD-A1EF-01A
	TCGA-BC-A10S-01A	TCGA-CC-A3MB-01A
	TCGA-5C-A9VG-01A	TCGA-DD-AAD6-01A
	TCGA-DD-AAE4-01A	TCGA-G3-A25X-01A
	TCGA-2Y-A9H0-01A	TCGA-DD-A11C-01A
	TCGA-RC-A7SF-01A	TCGA-DD-A73A-01A
	TCGA-MI-A75H-01A	TCGA-DD-AAEG-01A
	TCGA-DD-A3A0-01A	TCGA-DD-AADO-01A
	TCGA-BC-A10Y-01A	TCGA-FV-A3I1-01A
	TCGA-DD-AAVQ-01A	TCGA-CC-5260-01A
	TCGA-DD-AACW-01A	TCGA-LG-A9QD-01A

	TCGA-CC-A9FV-01A	TCGA-BC-A69I-01A
	TCGA-ZP-A9CV-01A	TCGA-DD-AAEK-01A
	TCGA-DD-AAVV-01A	TCGA-UB-A7MA-01A
	TCGA-UB-A7MC-01A	TCGA-DD-AACJ-01A
	TCGA-DD-AADG-01A	TCGA-DD-AADV-01A
	TCGA-MI-A75I-01A	TCGA-BC-A10R-01A
	TCGA-FV-A2QQ-01A	TCGA-DD-AACT-01A
	TCGA-ED-A8O5-01A	TCGA-RC-A7S9-01A
	TCGA-RC-A6M4-01A	TCGA-DD-AAVY-01A
	TCGA-BC-A10X-01A	TCGA-DD-A4NR-01A
	TCGA-2Y-A9H6-01A	TCGA-DD-A118-01A
	TCGA-MR-A520-01A	TCGA-KR-A7K0-01A
	TCGA-BC-A8YO-01A	TCGA-XR-A8TG-01A
	TCGA-2Y-A9GS-01A	TCGA-RC-A7SK-01A
	TCGA-NI-A8LF-01A	TCGA-DD-AADL-01A
	TCGA-G3-AAUZ-01A	TCGA-FV-A495-01A
	TCGA-DD-AACO-01A	TCGA-G3-A7M7-01A
	TCGA-G3-A3CI-01A	TCGA-DD-AAW2-01A
	TCGA-DD-AAD1-01A	TCGA-DD-AAC9-01A
	TCGA-DD-AAE3-01A	TCGA-DD-AACB-01A
	TCGA-DD-AACZ-01A	TCGA-BW-A5NO-01A
	TCGA-DD-AADB-01A	TCGA-G3-A25Z-01A
	TCGA-DD-A73D-01A	TCGA-BW-A5NQ-01A
	TCGA-BD-A3ER-01A	TCGA-G3-A25V-01A
	TCGA-DD-AADE-01A	TCGA-G3-A3CG-01A
	TCGA-XR-A8TF-01A	TCGA-DD-AADU-01A
**The accession number of DNA methylation profiles of 50 adjacent nontumor samples**	TCGA-EP-A26S-11A	TCGA-DD-A3A3-11A
	TCGA-DD-A11B-11A	TCGA-BC-A110-11A
	TCGA-DD-A1EB-11A	TCGA-DD-A116-11A
	TCGA-DD-A39W-11A	TCGA-BC-A10Y-11A
	TCGA-DD-A1EJ-11A	TCGA-BC-A10W-11A
	TCGA-DD-A3A2-11A	TCGA-EP-A12J-11A
	TCGA-DD-A39V-11A	TCGA-DD-A1E9-11A
	TCGA-DD-A11C-11A	TCGA-DD-A39Z-11A
	TCGA-DD-A1EH-11A	TCGA-DD-A1EF-11A
	TCGA-DD-A1EG-11A	TCGA-DD-A114-11A
	TCGA-BD-A2L6-11A	TCGA-DD-A1EE-11A
	TCGA-DD-A1EC-11A	TCGA-BD-A3EP-11A
	TCGA-DD-A1EI-11A	TCGA-ES-A2HT-11A
	TCGA-G3-A25X-11A	TCGA-DD-A39X-11A
	TCGA-BC-A112-11A	TCGA-BC-A10U-11A
	TCGA-BC-A10Z-11A	TCGA-DD-A11A-11A
	TCGA-G3-A25W-11A	TCGA-BC-A10T-11A

	TCGA-BC-A10Q-11A	TCGA-DD-A119-11A
	TCGA-DD-A1ED-11A	TCGA-FV-A2QR-11A
	TCGA-FV-A23B-11A	TCGA-DD-A3A1-11A
	TCGA-DD-A113-11A	TCGA-BC-A10X-11A
	TCGA-BC-A216-11A	TCGA-DD-A1EL-11A
	TCGA-BC-A10R-11A	TCGA-ES-A2HS-11A
	TCGA-BC-A10S-11A	TCGA-DD-A118-11A
	TCGA-DD-A11D-11A	TCGA-DD-A115-11A

Table S2. Primers for DNA methylation.

CpG	Primer name	Primer sequence
**SPP1-cg15460348**	cg15460348-F	GTTGGGTAGTGGTAGAAAATTTTATGAT
	cg15460348-Rbio	AACTAAATACACAACCCAATAACAAACT
	cg15460348-S	GGAGGAGGTAGAGTA
**LCAT-cg01817009**	cg01817009-F	AGTTGAGTTGAGGTTTGATTTTTTTAATAA
	cg01817009-Rbio	CCATTCCAACCCTAATACCTAC
	cg01817009-S	TTGGAGTTAAGAGAAGAAGG

Table S3. Primers for real-time PCR.

Gene	Forward (5' to 3')	Reverse (5' to 3')
**GAPDH**	TGACTTCAACAGCGACACCCA	CACCCTGTTGCTGTAGCCAAA
**SPP1**	TTTCGCAGACCTGACATCCA	TCCTCGCTTTCCATGTGTGA
**LCAT**	GCTCCATCAAGCCCATGCT	TCAGCTTGATGCTGGACATGA

Table S4. MethylMix models for DNA methylation-driven genes.

Gene	Normal mean DNA methylation	Tumor mean DNA methylation	Log FC	P value	Adjust P value	Pearson correlation	Pearson correlation P value
ANGPTL7	0.598501	0.894349	0.579483	4.15E-27	9.42E-25	-0.67466	1.35E-50
TGIF2LX	0.501591	0.236446	-1.085	3.49E-25	7.92E-23	-0.34723	5.95E-12
ODAM	0.906667	0.73878	-0.29543	3.09E-24	7.01E-22	-0.51104	4.50E-26
GPR182	0.708214	0.813501	0.199958	3.23E-24	7.32E-22	-0.33924	1.92E-11
FAM99A	0.455835	0.602568	0.402611	5.74E-23	1.30E-20	-0.43279	2.28E-18
JDP2	0.216939	0.321226	0.566295	8.89E-23	2.02E-20	-0.43343	2.01E-18

MAGEC1	0.703253	0.466534	-0.59206	3.08E-21	7.00E-19	-0.43564	1.29E-18
DKK3	0.200757	0.3756	0.903744	5.00E-21	1.14E-18	-0.44261	3.14E-19
UCHL1	0.342469	0.485864	0.504577	8.44E-21	1.92E-18	-0.3424	1.21E-11
LINC00898	0.768676	0.490118	-0.64925	1.89E-20	4.28E-18	-0.3682	2.35E-13
TRIM17	0.193816	0.338073	0.802649	9.28E-20	2.11E-17	-0.41119	1.44E-16
C1QTNF3	0.654725	0.750183	0.196354	1.61E-19	3.65E-17	-0.43516	1.42E-18
COX7B2	0.301777	0.184913	-0.70663	1.91E-19	4.33E-17	-0.47846	1.27E-22
MAGEB16	0.680121	0.564756	-0.26816	2.26E-19	5.13E-17	-0.32649	1.16E-10
NAA11	0.555313	0.396814	-0.48484	2.80E-19	6.35E-17	-0.33419	3.95E-11
MKRN3	0.658892	0.459853	-0.51887	2.83E-19	6.42E-17	-0.70003	6.47E-56
CSAG1	0.738375	0.574815	-0.36126	8.55E-19	1.94E-16	-0.37288	1.11E-13
KRBA1	0.534854	0.621566	0.216761	2.08E-18	4.72E-16	-0.34984	4.03E-12
GSTM5	0.411439	0.544014	0.402966	2.25E-18	5.10E-16	-0.46833	1.27E-21
BEND6	0.09519	0.26153	1.4581	2.42E-18	5.50E-16	-0.51449	1.85E-26
RNF17	0.692757	0.543301	-0.3506	2.89E-18	6.56E-16	-0.44649	1.41E-19
HIST1H2BH	0.146557	0.43179	1.55887	5.70E-18	1.29E-15	-0.36882	2.13E-13
MAGEA8	0.727395	0.560352	-0.37641	1.01E-17	2.29E-15	-0.32102	2.44E-10
LINC00944	0.417986	0.271015	-0.62508	1.04E-17	2.36E-15	-0.37106	1.49E-13
LCAT	0.532415	0.656223	0.301636	1.40E-17	3.18E-15	-0.64571	3.81E-45
LINC00200	0.436666	0.319289	-0.45167	2.67E-17	6.07E-15	-0.32334	1.78E-10
SLC17A8	0.415919	0.578276	0.475457	4.71E-17	1.07E-14	-0.32409	1.61E-10
ENTPD3	0.330597	0.516098	0.642571	1.13E-16	2.57E-14	-0.36158	6.69E-13

STMN3	0.080743	0.211193	1.387146	1.31E-16	2.96E-14	-0.49185	5.38E-24
ANKRD33	0.390602	0.493575	0.337568	1.88E-16	4.26E-14	-0.46628	2.00E-21
ADAM32	0.318046	0.497486	0.645419	2.73E-16	6.20E-14	-0.44481	2.00E-19
TCTEX1D1	0.235818	0.384072	0.703701	7.32E-16	1.66E-13	-0.33879	2.04E-11
LINC00879	0.893655	0.70652	-0.33899	1.25E-15	2.84E-13	-0.51063	5.01E-26
TUBA3C	0.41818	0.322818	-0.3734	2.20E-15	4.99E-13	-0.44215	3.45E-19
HIST3H2A	0.072431	0.271865	1.908207	2.59E-15	5.88E-13	-0.63643	1.61E-43
XAGE5	0.620743	0.479322	-0.373	3.29E-15	7.47E-13	-0.52774	5.44E-28
DDX53	0.511247	0.33817	-0.59627	3.87E-15	8.79E-13	-0.5252	1.08E-27
FITM1	0.351238	0.455706	0.375657	4.40E-15	1.00E-12	-0.54736	2.21E-30
NOVA1	0.080724	0.160377	0.990408	6.31E-15	1.43E-12	-0.44724	1.21E-19
RFPL4B	0.806643	0.681103	-0.24406	1.60E-14	3.63E-12	-0.44612	1.52E-19
SYNGR3	0.219426	0.54137	1.302884	1.92E-14	4.36E-12	-0.37671	5.92E-14
PES1P1	0.611819	0.399132	-0.61624	2.04E-14	4.64E-12	-0.55678	1.37E-31
PDK4	0.583225	0.783482	0.425848	2.05E-14	4.66E-12	-0.44225	3.38E-19
MT1M	0.238817	0.327249	0.454488	6.18E-14	1.40E-11	-0.38431	1.67E-14
PNMA5	0.798526	0.672379	-0.24807	1.05E-13	2.39E-11	-0.45809	1.20E-20
MT3	0.196294	0.322219	0.715026	2.54E-13	5.77E-11	-0.33453	3.76E-11
DYDC1	0.23868	0.358606	0.587318	4.04E-13	9.18E-11	-0.39185	4.59E-15
LINC01093	0.357267	0.48761	0.448726	4.51E-13	1.02E-10	-0.6133	1.05E-39
S100A10	0.335772	0.221842	-0.59795	5.46E-13	1.24E-10	-0.60693	1.05E-38
AC126407.1	0.736504	0.599793	-0.29623	5.72E-13	1.30E-10	-0.32841	8.87E-11

KLHL35	0.263677	0.341921	0.374887	6.12E-13	1.39E-10	-0.41297	1.03E-16
GFRA3	0.534313	0.667395	0.320856	6.70E-13	1.52E-10	-0.47145	6.29E-22
PRDM9	0.420626	0.300465	-0.48534	1.47E-12	3.34E-10	-0.43057	3.54E-18
ZFPM2-AS1	0.796561	0.494947	-0.68651	2.80E-12	6.36E-10	-0.41821	3.86E-17
SALL4	0.584291	0.718151	0.2976	3.06E-12	6.95E-10	-0.33116	6.04E-11
SLITRK3	0.107373	0.193864	0.852406	3.97E-12	9.00E-10	-0.34587	7.28E-12
C5orf58	0.519425	0.401992	-0.36975	6.21E-12	1.41E-09	-0.43281	2.27E-18
RASL12	0.398482	0.555636	0.479625	1.05E-11	2.39E-09	-0.47505	2.78E-22
NAT2	0.635021	0.75559	0.250798	1.31E-11	2.97E-09	-0.59698	3.41E-37
CKMT2	0.448224	0.592035	0.401461	2.03E-11	4.61E-09	-0.54699	2.45E-30
GTSF1	0.562248	0.460025	-0.2895	2.57E-11	5.84E-09	-0.65595	5.25E-47
TNNC1	0.848651	0.709832	-0.25769	2.64E-11	5.99E-09	-0.64657	2.68E-45
LINC01139	0.30003	0.218458	-0.45775	4.46E-11	1.01E-08	-0.39101	5.31E-15
MAGEB1	0.72781	0.611517	-0.25117	6.95E-11	1.58E-08	-0.3409	1.51E-11
LRRC4	0.429583	0.538122	0.324998	6.36E-10	1.44E-07	-0.37757	5.13E-14
NSUN7	0.276594	0.432818	0.645988	1.03E-09	2.35E-07	-0.67917	1.67E-51
GSTM1	0.146627	0.292363	0.995615	1.40E-09	3.17E-07	-0.67576	8.09E-51
ZNF192P1	0.254025	0.364523	0.521041	2.16E-09	4.91E-07	-0.32011	2.76E-10
CYP2C9	0.437312	0.544959	0.317487	3.42E-09	7.76E-07	-0.6621	3.71E-48
SLC16A11	0.092765	0.085291	-0.12119	3.96E-09	8.98E-07	-0.41879	3.46E-17
MYCBPAP	0.383404	0.505397	0.398552	4.46E-09	1.01E-06	-0.37818	4.64E-14
ARHGAP10	0.225821	0.379768	0.749936	5.02E-09	1.14E-06	-0.33747	2.47E-11

ANKRD30A	0.648031	0.552116	-0.23109	5.37E-09	1.22E-06	-0.33653	2.83E-11
MT1E	0.077442	0.14722	0.926785	9.44E-09	2.14E-06	-0.48704	1.70E-23
CALCA	0.216619	0.300914	0.474192	1.77E-08	4.01E-06	-0.34436	9.09E-12
TINAG	0.478898	0.387916	-0.30398	2.58E-08	5.86E-06	-0.42165	2.01E-17
STAU2-AS1	0.85309	0.668534	-0.3517	3.32E-08	7.53E-06	-0.3448	8.52E-12
ZNF391	0.06984	0.168045	1.266723	3.91E-08	8.88E-06	-0.55346	3.69E-31
ZNF83	0.087857	0.157465	0.841808	4.88E-08	1.11E-05	-0.52165	2.81E-27
TMEM220	0.240233	0.286961	0.256425	4.93E-08	1.12E-05	-0.55621	1.62E-31
CDS1	0.102036	0.215762	1.080364	8.89E-08	2.02E-05	-0.4813	6.57E-23
ESYT3	0.158918	0.215606	0.440115	1.01E-07	2.29E-05	-0.34426	9.22E-12
P3H4	0.679843	0.733163	0.108931	1.42E-07	3.23E-05	-0.44547	1.74E-19
EFNA5	0.055641	0.129111	1.214406	1.65E-07	3.75E-05	-0.34631	6.82E-12
ADAD1	0.69508	0.629793	-0.1423	2.67E-07	6.05E-05	-0.36899	2.07E-13
AC004687.1	0.611795	0.664017	0.118171	2.90E-07	6.57E-05	-0.52703	6.60E-28
NRBF2P2	0.921541	0.814751	-0.17769	2.91E-07	6.61E-05	-0.37565	7.04E-14
ZNF233	0.208604	0.325114	0.640179	4.19E-07	9.51E-05	-0.51179	3.72E-26
SLC22A31	0.159581	0.252618	0.662668	5.88E-07	0.000133	-0.47011	8.51E-22
GNG4	0.158714	0.269295	0.762754	7.23E-07	0.000164	-0.44282	3.00E-19
LAMA4	0.548516	0.601644	0.133377	1.03E-06	0.000234	-0.35988	8.72E-13
BATF	0.486851	0.552633	0.182843	1.08E-06	0.000245	-0.52157	2.86E-27
LINC00482	0.149412	0.104104	-0.52127	1.31E-06	0.000297	-0.33246	5.04E-11
EPHA6	0.106792	0.173673	0.70157	2.25E-06	0.000511	-0.33381	4.16E-11

CTHRC1	0.227724	0.375331	0.720878	2.49E-06	0.000565	-0.4043	5.06E-16
INHBC	0.487212	0.623443	0.355708	2.92E-06	0.000663	-0.44539	1.77E-19
RARRES2P6	0.733575	0.535315	-0.45456	3.13E-06	0.00071	-0.3287	8.52E-11
GULP1	0.185852	0.250788	0.432314	3.43E-06	0.00078	-0.34539	7.82E-12
ZNF382	0.105092	0.258652	1.299361	4.91E-06	0.001114	-0.70833	8.90E-58
PLEK2	0.26863	0.358336	0.41569	5.54E-06	0.001257	-0.4536	3.15E-20
LUZP4	0.797734	0.664838	-0.2629	5.82E-06	0.001322	-0.32171	2.22E-10
SSX1	0.848815	0.686323	-0.30656	6.12E-06	0.001389	-0.67152	5.63E-50
RBP2	0.729655	0.656266	-0.15293	6.96E-06	0.001579	-0.32045	2.64E-10
EPCAM	0.298165	0.346537	0.216898	8.57E-06	0.001945	-0.38196	2.47E-14
PON3	0.116791	0.106157	-0.13772	9.24E-06	0.002097	-0.35143	3.18E-12
HIST1H2AE	0.051308	0.09791	0.932258	1.17E-05	0.002665	-0.38395	1.77E-14
PRSS3	0.299422	0.380606	0.346117	1.56E-05	0.003535	-0.56931	2.97E-33
RPS6KA6	0.185734	0.286761	0.626612	1.67E-05	0.003781	-0.45695	1.54E-20
C19orf33	0.480451	0.539113	0.166199	1.77E-05	0.004009	-0.59168	2.07E-36
G0S2	0.121895	0.108545	-0.16735	2.32E-05	0.00526	-0.35062	3.59E-12
CLGN	0.046231	0.070765	0.614178	2.49E-05	0.00565	-0.40385	5.49E-16
B3GALNT1	0.295373	0.371333	0.330173	2.52E-05	0.005712	-0.52844	4.51E-28
PLEKHS1	0.636129	0.662754	0.059155	2.60E-05	0.005903	-0.56951	2.79E-33
MAT1A	0.205798	0.179779	-0.19501	3.96E-05	0.008984	-0.54405	5.73E-30
NRSN2	0.216029	0.292712	0.438256	4.98E-05	0.011298	-0.8062	3.98E-86
PPP1R14BP2	0.271942	0.35202	0.372362	5.53E-05	0.012557	-0.51155	3.96E-26

IGF1	0.254084	0.354066	0.478714	5.72E-05	0.012995	-0.71868	3.39E-60
TM4SF19	0.654345	0.590597	-0.14788	6.10E-05	0.013839	-0.46573	2.26E-21
PAX8	0.640223	0.694305	0.116995	6.19E-05	0.014059	-0.56212	2.73E-32
TAT	0.254916	0.313219	0.297146	7.34E-05	0.016655	-0.69102	5.79E-54
FBP1	0.088292	0.133794	0.599663	0.000108	0.024544	-0.47534	2.60E-22
ABCD1	0.349034	0.287752	-0.27854	0.000147	0.03337	-0.39084	5.47E-15
SPP1	0.525435	0.426909	-0.29959	0.000148	0.033621	-0.6959	5.20E-55

Table S5. Times of nonzero coefficients of 51 prognostic genes in 1000 LASSO analyses.

**Gene**	**Times**
SPP1	1000
LCAT	1000
CYP2C9	993
SLC16A11	977
LINC00200	872
GPR182	872
ZFPM2-AS1	740
AC004687.1	740
ANKRD33	585
TM4SF19	585
TAT	420
GSTM5	420
MT3	420
S100A10	420
B3GALNT1	247
ADAM32	11
ADAD1	1
LRRC4	1
FAM99A	0
CTHRC1	0
LINC01139	0
TMEM220	0
RNF17	0
PON3	0
KRBA1	0
PRDM9	0

PDK4	0
MAT1A	0
LINC00879	0
TGIF2LX	0
IGF1	0
ESYT3	0
TINAG	0
C5orf58	0
EFNA5	0
LINC01093	0
FITM1	0
CSAG1	0
XAGE5	0
GULP1	0
RFPL4B	0
MAGEA8	0
EPHA6	0
TUBA3C	0
PES1P1	0
FBP1	0
INHBC	0
ZNF391	0
CDS1	0
UCHL1	0
NAA11	0

Table S6. Correlation between the CpGs methylation in promoters and corresponding gene expression in the TCGA validation dataset.

CpG methylation	Gene expression	Pearson correlation	P value
cg15460348	SPP1	-0.742097867	< 0.001
cg20261167	SPP1	-0.713492599	< 0.001
cg00088885	SPP1	-0.398463823	< 0.001
cg00583003	SPP1	-0.103088098	0.047267673
cg01817009	LCAT	-0.78326538	< 0.001
cg26924825	LCAT	-0.677728846	< 0.001
cg07207367	LCAT	-0.610172793	< 0.001
cg06134860	LCAT	-0.6058284	< 0.001
cg05233094	LCAT	-0.570366149	< 0.001
cg02564259	LCAT	-0.539081443	< 0.001
cg09581611	LCAT	-0.51464855	< 0.001
cg06839111	LCAT	-0.460697425	< 0.001
cg09495207	LCAT	-0.44978721	< 0.001
cg00594148	LCAT	-0.134918938	0.009315519

cg01489608	LCAT	-0.098677906	0.057604233
cg07599484	LCAT	-0.065952667	0.204899923
cg08944929	LCAT	-0.037843008	0.467227055
